# Exploration of Antifungal and Immunomodulatory Potentials of a Furanone Derivative to Rescue Disseminated Cryptococosis in Mice

**DOI:** 10.1038/s41598-017-15500-8

**Published:** 2017-11-13

**Authors:** Sudarshan Singh Rathore, Muthukrishnan Isravel, Sridharan Vellaisamy, David Raj Chellappan, Lalitha Cheepurupalli, Thiagarajan Raman, Jayapradha Ramakrishnan

**Affiliations:** 1 Actinomycetes Bioprospecting Lab, Centre for Research in Infectious Diseases (CRID), School of Chemical and Biotechnology, SASTRA University, Tirumalaisamudram, Thanjavur, Tamilnadu 613401 India; 2 Department of Chemistry, School of Chemical and Biotechnology, SASTRA University, Tirumalaisamudram, Thanjavur, Tamilnadu 613401 India; 3 Central Animal Facility (CAF), School of Chemical and Biotechnology, SASTRA University, Tirumalaisamudram, Thanjavur, Tamilnadu 613401 India; 4 Department of Advanced Zoology and Biotechnology, Ramakrishna Mission Vivekananda College, Mylapore, Chennai 600004 India; 5 Department of Chemistry and Chemical Sciences, Central University of Jammu, Rahya-Suchani (Bagla), Samba Jammu, J&K 181143 India

## Abstract

*Cryptococcus neoformans* infection is quite complex with both host-pathogen interaction and host immune profile determining disease progress and therapeutic outcome. Hence in the present study, the potential utility of (*E*)-5-benzylidenedihydrofuran-2(3 *H*)-one (compound-**6**) was explored as an effective anticryptococcal compound with immunomodulatory potential. The efficacy of compound-**6** in pulmonary cryptococosis model using H99 strain was investigated. The effective dose was found to provide 100% survival, with a significant reduction of yeast burden in lungs and brain. The biodistribution analysis provided evidence for the presence of higher concentration of compound-**6** in major organs including lungs and brain. In addition, compound-**6** treated mice had significantly higher expression of IL-6, IL-4 and IFN-γ in lung and brain. Similarly, elevated expression of TNF-α, IL-β1 and IL-12 were observed in lungs, suggesting the protective host response against *C. neoformans*. The reduction and clearance of fungal load in systemic organs and mouse survival are notable results to confirm the ability of compound-**6** to treat cryptococcosis. In conclusion, the low molecular weight (174 Da), lipophilicity, its ability to cross blood brain barrier, and facilitating modulation of cytokine expression are the added advantages of compound-**6** to combat against disseminated cryptococosis.

## Introduction

*Cryptococcus neoformans* is a major opportunistic yeast pathogen causing pulmonary cryptococcosis and fatal cryptococcal meningitis (CM) in immunocompromised individuals, particularly in HIV infected patients^1,2^. An updated global annual incidence of CM was estimated to be 223100 with more than 80% mortality in patients with non-protective immune responses^3^. Albeit being declared as a growing problem worldwide by Centre for Disease Control of the United States, research in combating CM remains in its infancy^4^. The severity of the disease is due to multiple virulence factors of *C. neoformans* such as polysaccharide capsule, melanin pigment production, phospholipases, mannitol, urease, and proteinase production^5,6^. Presence of capsule is the most important among these, as it is primarily responsible for resisting phagocytosis and triggers non-protective immune responses^7–10^. Similarly, melanization protects yeast cells against antifungal drugs^11,12^. Melanin and the polysaccharide capsules act as antifungal resistance factors leading to disseminated cryptococosis.

The current standard therapy for cryptococcal infection include amphotericin B (AmpB) in conjunction with flucytosine as induction therapy^13^. The other effective combination is AmpB deoxycholate (0.7–1.0 mg/kg) combined with flucytosine (100 mg/kg/day for 2 weeks), followed by further consolidation therapy with fluconazole^14,15^. However, fluconazole resistance is an emerging problem and prolonged medication can lead to other yeast infections. Protein abnormalities can persist for years producing adverse effects such as nephrotoxicity; side effects of prolonged antibiotic therapy^16,17^. In addition, the availability of flucytosine is an important issue in Africa and Asia where CM is more common^18^. Lipid based formulation of AmpB has excellent efficiency as it has better bioavailability across the blood-brain barrier as well as reduced nephrotoxicity. This is an exciting option for those who can afford it but the majority of CM patients cannot afford the liquid formulation, being in the low and middle income groups^19,20^. Moreover, in the last 30 years, echinocandins are the only new class of antifungal drugs that has been developed but with no activity against *C. neoformans* ^21,22^. Hence, there is definitely an urgent need for new drugs that are effective against CM and host friendly.

In terms of alternative treatment approaches as far as immunocompromised individuals are concerned, immunotherapy, passive immunization and cytokine-based approaches are gaining prominence. These strategies have one important consequence, immunomodulation. Monoclonal antibodies have been used for passive immunization against capsule^23,24^. This led to phase I trial with the monoclonal antibody 18B7 in HIV patients against CM^25^. Another monoclonal antibody 2G8, targeted against cell wall glucan was also shown to be effective^26^. However, antibody-based immune therapies are complex procedures, expensive and in some cases, can produce toxicity. Cytokine-therapy has also been gaining importance for cryptococosis treatment, which was successful in experimental animals, but it is contradictory in clinical trials. For instance, in rodents, use of IFN-γ has shown good results against systemic cryptococcosis^27^. The same was also tested successfully in one human patient with CM^28^. However, one issue with IFN-γ usage is that it reduces anticryptococcal activity of human macrophages, hence its use in humans is not conclusive^28^. Moreover, the *in vivo* efficacy of any immunomodulation might change depending on the stage and site of infection. The use of Th1 cytokines TNF-α, IFN-γ, IL-12, IL-17, IL-23 and Th2 cytokines IL-4, IL-10, IL-13 has shown some contradictory results^29^. Thus, an antifungal agent that modulates the host immune responses will significantly improve the outcomes of CM.

Furanone and its derivatives have been widely isolated from plants, bacteria, fungi, algae and actinomycetes^30,31^. Due to the presence of rigid five-membered ring structure with least complexity, furanone derivatives are extensively being investigated as potential skeleton to treat diverse diseases^30–33^. Natural and synthetic furanones were extensively investigated for its antibacterial, antiquorum sensing and antibiofilm property^30,32,34^, however the potential utility of furanone compound to treat cryptococcal infection was not explored. In our previous study, during the investigation on adaptive laboratory evolution of *Streptomyces* sp, the Gas Chromatography-Mass Spectrum (GC-MS) profiling of the evolved strain revealed the presence of dihydro-3,3-dimethyl-2(3 H)-furanone with good antagonistic activity against *C. neoformans* ^35^. Hence it was of interest to synthesize furanone derivatives with 5 positions, (*E*)-5-benzylidenedihydrofuran-2(3 *H*)-one, that have shown immense potential against 7 different strains of *C. neoformans*, including H99 strain. The study involves *in vivo* approaches in exploring the potential usage of the proposed compound for the effective management of cryptococcal infection. Parallely, we studied the ability of the compound to reach target organs and subsequently efficacy in fungal clearance was analysed. Furthermore, the influence of compound**-6** on TLR-2, Th1 and Th2 responses in lung and brain tissues was studied. To the best of our knowledge, this study is the first report showing the antagonistic and immunomodulation of furanone compound, (*E*)-5-benzylidenedihydrofuran-2(3 *H*)-one against *C. neoformans* in a murine model and therefore valuable for highlighting the potential use of furanone for the effective management of disseminated cryptococosis.

## Results

### Minimum inhibitory concentration (MIC) of compound-6 against various strains of *C. neoformans*

MIC_0.5_ and MIC_0.9_ of compound-**6** against various strains of *C. neoformans* were 400 µg/mL and 600 µg/mL, respectively (Table 1). MIC_0.9_ of compound-**6** (600 µg/mL) against H99 strain was higher than AmpB (10.7 µg/mL). A similar sensitivity pattern was observed for *Candida albicans* against compound**-6**. No activity was observed for compound**-11** against any strain of *C. neoformans* and *C. albicans*. The reduction of yeast count with corresponding MIC values was observed in potato dextrose agar (PDA) plates (Supplementary Fig. S1).

**Table 1:** MIC of Compound-6 and AmpB against different strains of *C. neoformans* and *Candida albicans*.

Strain	Concentration (μg/mL)			
	Compound-6		AmpB	
	MIC_0.5_	MIC_0.9_	MIC_0.5_	MIC_0.9_
***C. neoformans***				
H99	400	600	10.7	10.7
BAC21	200	300	2.7	8.0
CDC103	100	400	2.7	5.3
RPC29	300	500	5.3	8.0
RPC30	300	500	2.7	8.0
ATCC 14116	200	400	2.7	8.0
Clinical strain	300	400	5.3	8.0
***Candida albicans***				
Clinical strain	400	600	10.7	13.3

### *In vitro* cytotoxicity assay

**Figure 1:**
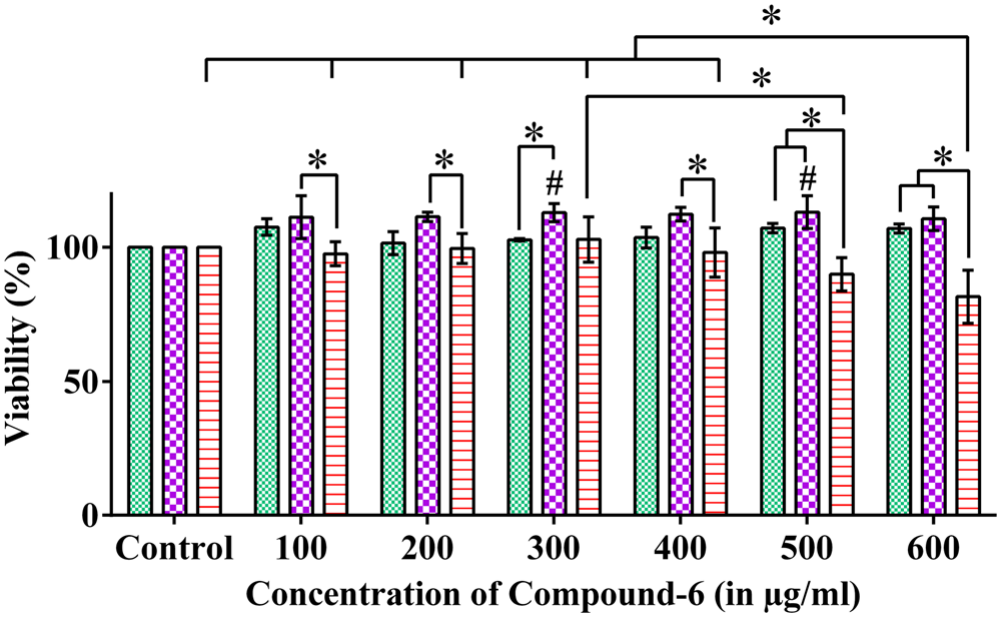
*In vitro* cytotoxicity assay of compound-6. In HepG2 cell line, more than 80% viability was observed up to 600 µg/mL at 24 hours incubation. ^#^And *represents p ≤ 0.05 from control and other variables respectively; comparison by two-way ANOVA followed by Tukey’s multiple comparisons test (Supplementary Table S1) and graphs were obtained using GraphPad Prism software.

As the MIC_0.9_ of compound-**6** was 600 µg/mL for different strains of *C. neoformans*, we then proceeded to check the *in vitro* toxicity assay. From Fig. 1 and Supplementary Fig. S2(I), it was evident that more than 80% and 100% cell viability were recorded for HepG2 and J774.A.1 cell lines respectively up to 600 µg/mL at 24 hours incubation.

### *In vitro* immunomodulation study of compound-6

Having shown the antifungal activity of compound-**6**, we then proceeded to explore its potential as an immunomodulant. In this, a series of *in vitro* experiments were performed with macrophages that were either treated with lipopolysaccharide (LPS) with IFN-γ or compound-**6** or left untreated. At the end of incubation gene expression analyses of various cytokines were performed. Compound-**6** treated macrophages showed enhancement in the expression of TLR-2 (1.6-fold), IL-β1 (5.2-fold), TNF-α (4-fold), IFN-γ (0.8-fold) and IL-12 (0.3-fold) (Supplementary Fig. S2(II)). On the other hand, both IL-10 and IL-4 showed down regulation, whereas IL-6 did not show any change. All these when compared to untreated macrophages. This was similar to the cytokine levels observed with LPS with IFN-γ treatment, except that the TNF-α, IFN-γ, IL-6 and IL-12 levels were much higher than compound-**6** treated macrophages. This effect of LPS with IFN-γ on macrophages was on expected lines. However, the compound produced a significant increase in proinflammatory cytokines and inhibited anti-inflammatory cytokines, like LPS with IFN-γ, and this shows the potency of the compound-**6** to produce immunomodulation, primarily TLR-2 related responses, even in the absence of the pathogen, whereas LPS with IFN-γ is a well known antigen.

### Acute toxicity study

**Figure 2:**
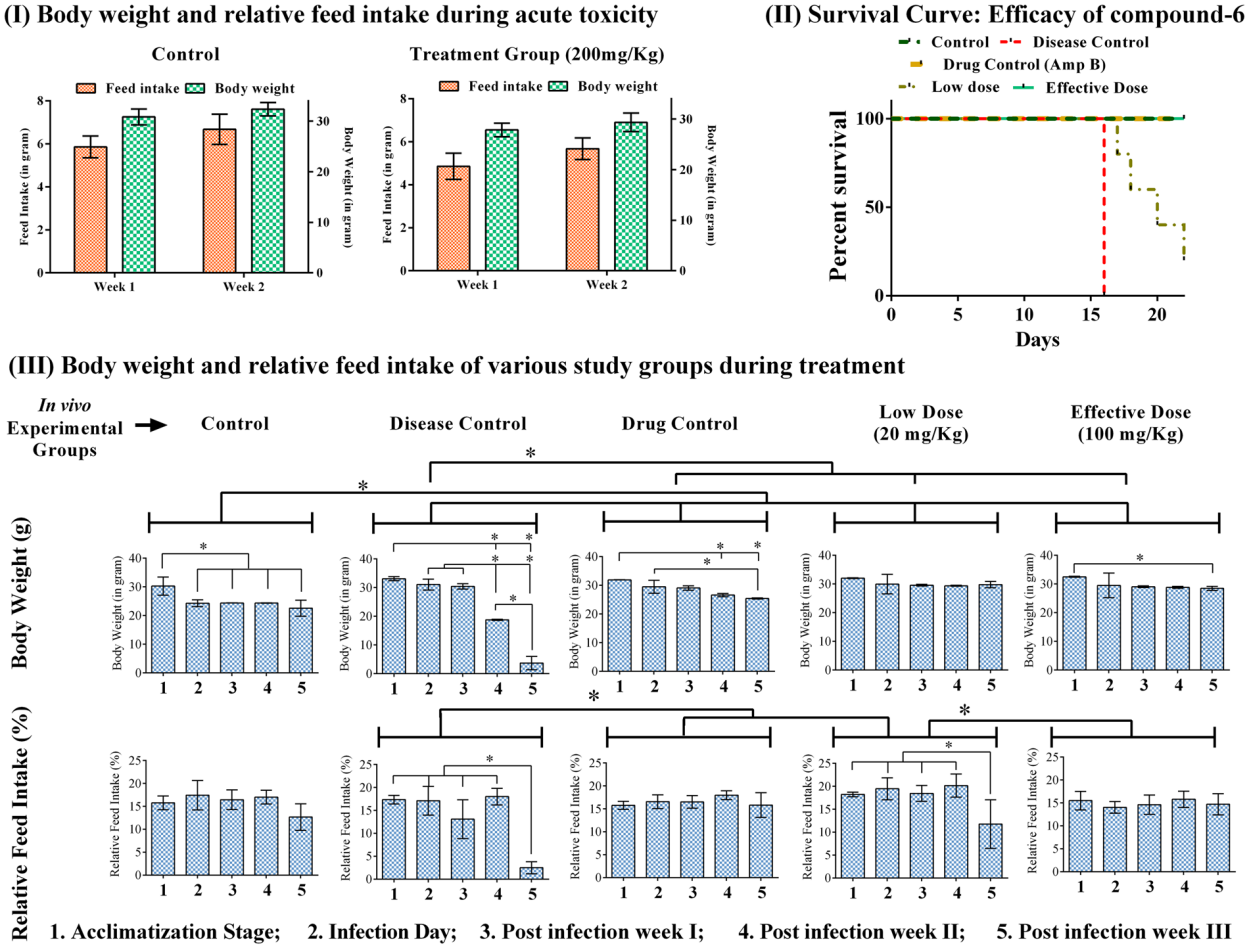
(**I**) Acute *in vivo* toxicity of compound-6 at 200 mg/kg given in a single dose. Animals (n = 6) were administered iv with compound-**6** and were monitored for 14 days. No toxic effect or mortality was observed at 200 mg/kg (500X MIC). Body weight and feed intake were normal throughout the study. (**II**) Efficacy of compound-6 in H99 infected mice: Infected mice were treated with compound-**6** (iv; Low dose = 20 mg/kg/day and Effective dose = 100 mg/kg/day), AmpB (iv; 0.5 mg/kg/day). Control and disease control group were injected with 1% DMSO for 21 days. 100% survival was obtained in effective dose treatment group similar to that of control and drug control. 20% survival was observed in low dose treated group. Disease control group was displayed with 100% mortality from 16^th^ day of post infection. (**III**) Body weight and relative feed intake of various study groups during treatment. The body weight and feed consumption were monitored during acclimatization and post infection period of 22 days. There was no significant difference in body weight and relative feed intake in effective dose group, which was comparable to that of control. Whereas, in case of disease control, reduction in body weight and feed intake was observed from 2^nd^ and 3^rd^ week onwards. Bar represents mean and SD for 6 animals. *Represents p ≤ 0.05; comparison by two-way ANOVA followed by Tukey’s multiple comparisons test (Supplementary Table S3) and graphs were obtained using GraphPad Prism software.

During acute toxicity study, the effect of compound-**6** on mice survival and general behavioral pattern were recorded. No toxic symptoms or mortality were observed in any animal after the intravenous administration of compound-**6** at a single dose level of 200 mg/kg body weight (500X MIC). 100% animal survival was noticed till the end of the study period. The animals were normal and displayed no significant changes in behavior, sleep and breathing. Furthermore, the consumption of food and water by the animals were normal throughout the study. No significant variation in weight was recorded in animals challenged with compound-**6** (Fig. 2(I)).

### Therapeutic effect of compound-6 in rescuing mice from H99 infection

#### Survival rates of treated mice

Mice of all the groups were monitored throughout the study including acclimatization and post infection period and deaths were recorded. Compound-**6**-100 mg/kg/day was more effective in rescuing the mice from H99 infection with 100% survival. The survival rates of compound-**6**-100 mg/kg/day group were comparable to that of AmpB. The improvement in survival rate was also observed in compound-**6**-20 mg/kg/day group with 20% survival, in which mortality was observed from 17^th^ day post infection. The untreated control group showed 100% mortality from 8^th^ day post infection (Fig. 2(II)). Supportingly, there was a significant improvement in feed intake and body weight in the case of test and drug control (Fig. 2(III)), when compared to disease control.

When we compared the efficacy of two different doses of compound-**6** (20 and 100 mg/kg/day) in improving mice survival (Fig. 2(II)), it was observed that 100 mg/kg/day was more effective in reducing fungal load and produced improvement in mice survival. Hence hereafter in the manuscript, we will refer to compound-**6** at 20 mg/kg/day as low dose and 100 mg/kg/day as effective dose.

### Penetration of antifungal agents into organs and reduction of fungal burden

**Figure 3:**
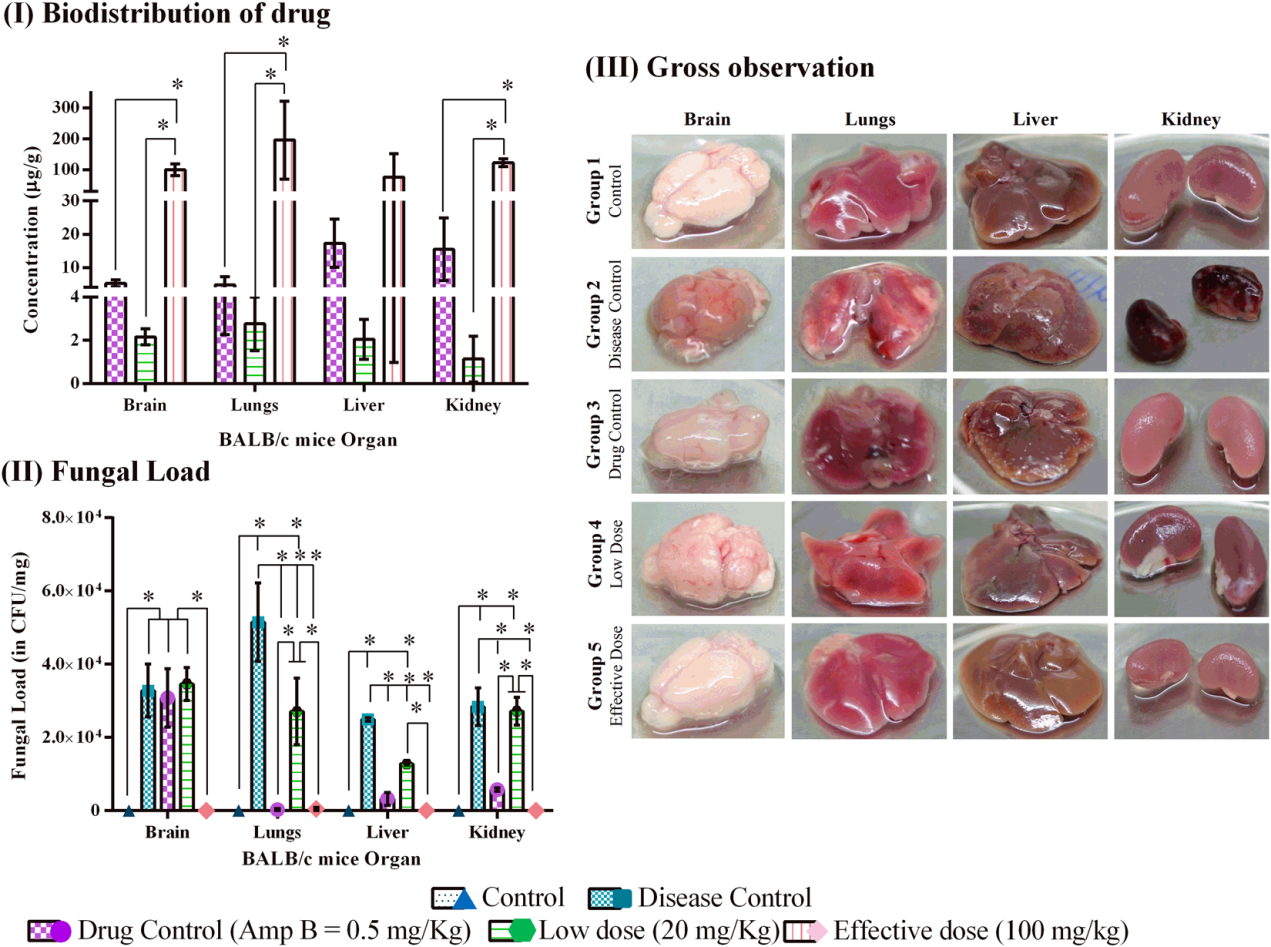
(**I**) Biodistribution of compound-6 and AmpB: Concentration of compound-**6** and AmpB in each organ was expressed in µg/g. (**II**) Fungal load: Fungal load was estimated in PDA plates. Absence of yeast colonies were observed in effective dose treatment group. (**III**) Gross observation of systemic organs of various study groups: Organ damage was observed in disease and low dose treated groups. Liver abnormality was recorded in AmpB treated. The organs were normal in effective dose treated group and were similar in appearance to that of control. Bar represents mean and SD for triplicates. *Represents p ≤ 0.05; comparison by One-way and two-way ANOVA followed by Tukey’s multiple comparisons test (Supplementary Table S4) and graphs were obtained using GraphPad Prism software.

After the treatment period, the collected organs were investigated for drug bioavailability and fungal burden. Figure 3(I and II) shows the correlation between the availability of drug in the target organs and subsequent reduction in fungal load. The mean concentration of effective dose in different organs ranged between 76.3–196.46 µg/g, with maximum availability in lungs (196.46 µg/g). Correspondingly, absence of yeast colonies in brain, kidney, liver and substantial reduction of fungal burden from 5 × 10^4^ to 5 × 10^2^ CFU was noticed in lungs (Fig. 3II). In case of low dose, drug concentration was very much less (1.13–2.75 µg/g) in all organs with ~48% reduction of fungal load in brain and lungs. The availability of AmpB in tissues ranged between 4.75–17.3 µg/g. Though AmpB concentration was less in all organs when compared to compound-**6**, the fungal reduction was achieved efficiently in all organs except brain which was found to have high fungal load similar to that of disease group Fig. 3(II).

### Gross observation of systemic organs

The gross observations of systemic organs of all groups are shown in Fig. 3(III). No changes were observed in gross morphology of control and effective dose treated group. In case of drug control (AmpB), a notable difference in liver was observed as white spots on the surface which was not recorded in any organs of any other groups. This might be due to the hepatotoxic nature of AmpB. Interestingly, kidney damage in disease group was observed. As of now limited published data is available on kidney involvement in cryptococossis^36^. Thus the pathophysiology of renal involvement in cryptococcosis needs to be addressed.

### Histopathology of pulmonary cryptococcosis

**Figure 4:**
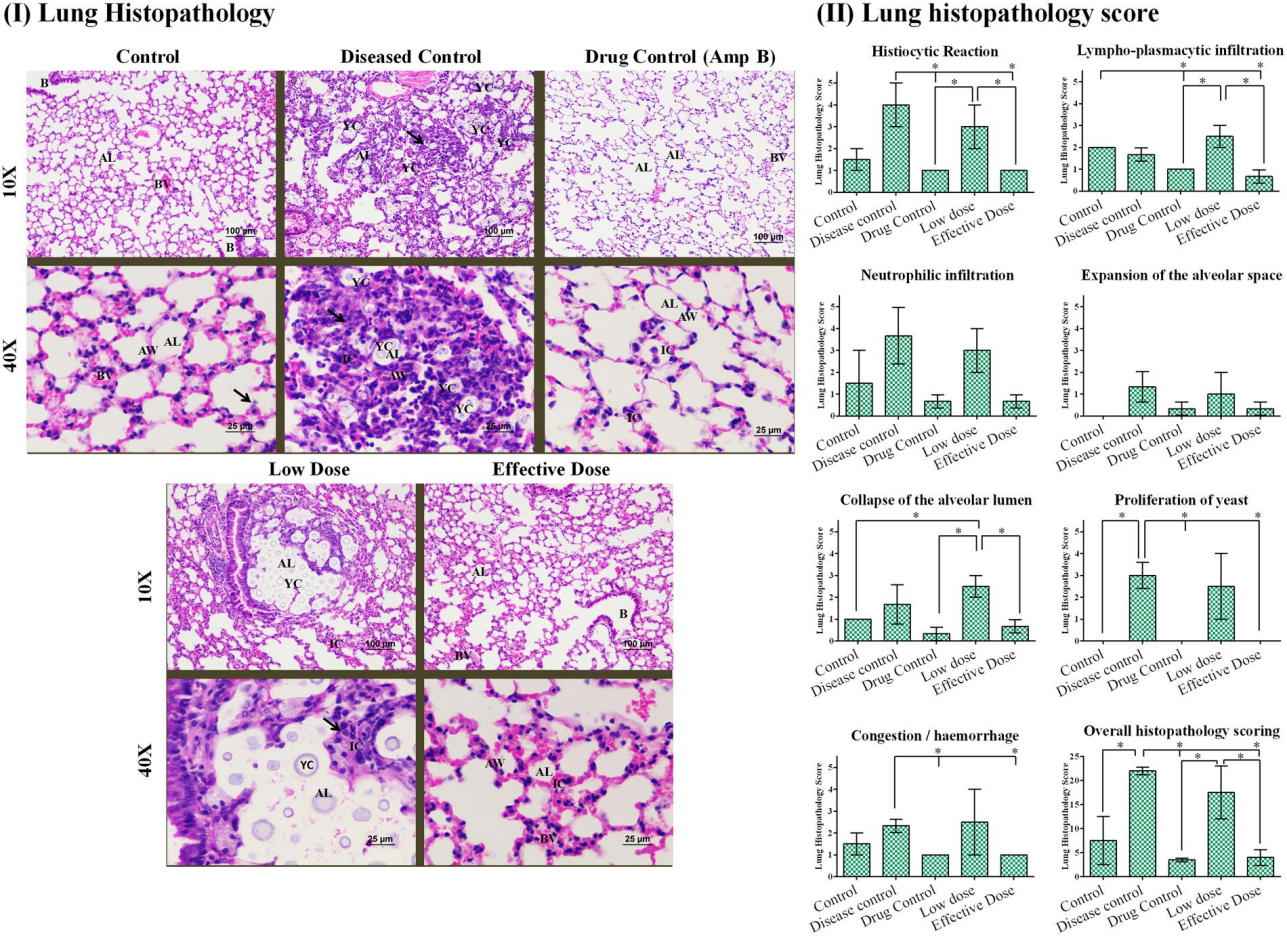
(**I**) Lung Histopathology: Lungs section of control group mice with normal alveolar wall (AW) and its capillaries. The pneumocytes, alveolar lumina (AL) and blood vessels (BV) were normal. Few alveolar macrophages (arrow), normally present in alveolar wall were observed. Bronchial (B) walls and their epithelial cells were normal. Disease control group mice with collapsed alveolar lumina (arrow); accumulation of numerous inflammatory cells (IC) in and around, alveolar lumina (AL) and alveolar wall (AW). Abundance of yeast cells (YC) in alveolar lumina (AL). Drug control group mice with normal alveolar walls; Inflamatory cells (IC) were normally present in alveolar wall (AW). Pulmonary blood vessel (BV) normally present. Absence of yeast cells in alveolar lumina (AL). Low dose group mice group with expansion of Alveolar lumina (AL); accumulation of inflamatory cells (IC) in and around, alveolar lumina and alveolar wall. Abundance of yeast cells (YC) in Alveolar lumina (AL). Effective dose group mice with minimal alveolar expansion and normal alveolar walls (AW); Inflamatory cells (IC) were normally present in alveolar wall (AW). Pulmonary blood vessel (BV) normally present. Absence of yeast cells in alveolar lumina (AL). Bronchial (B) walls and their epithelial cells were normal (H&E). (**II**) Lung histopathology score for BALB/c mice treated with compound-6. The histopathological parameters such as histiocytic reaction, lympho-plasmacytic infiltration, neutrophilic infiltration, expansion of the alveolar space, collapse of the alveolar lumen, proliferation of yeast and congestion/haemorrhage was observed for all the groups. Based on overall histopathology scoring, the disease control was marked with high score followed by low dose, control, drug control and effective dose. Bar represents mean and SD for duplicates. *Represents p ≤ 0.05; comparison by two-way ANOVA followed by Tukey’s multiple comparisons test (Supplementary Table S6) and graphs were obtained using GraphPad Prism software.

Pulmonary sections of mice infected with *C. neoformans* H99 revealed enlargement of the alveolar space as a result of moderate to marked yeast proliferation along with marked macrophage response, lympho-plasmacytic and neutrophilic infiltration in and around alveolar lumen and alveolar wall. In addition, minimal to mild collapse of alveolar lumen and damage of bronchial wall was observed when compared with uninfected group. In contrast, furanone treatment group was observed with striking difference in lung section with very minimal expansion of alveolar space and less macrophage response. The inflammatory reaction constituted by histiocytes, lympho-plasmacytes and neutrophiles was minimal which can be highly correlated with more reduction in yeast proliferation in alveolar space. The histopathologic characteristics and score (Fig. 4(I,II) and Supplementary Table S5) of effective dose was similar to that of Amb group (drug control).

### Cytokine expression in Lungs

**Figure 5:**
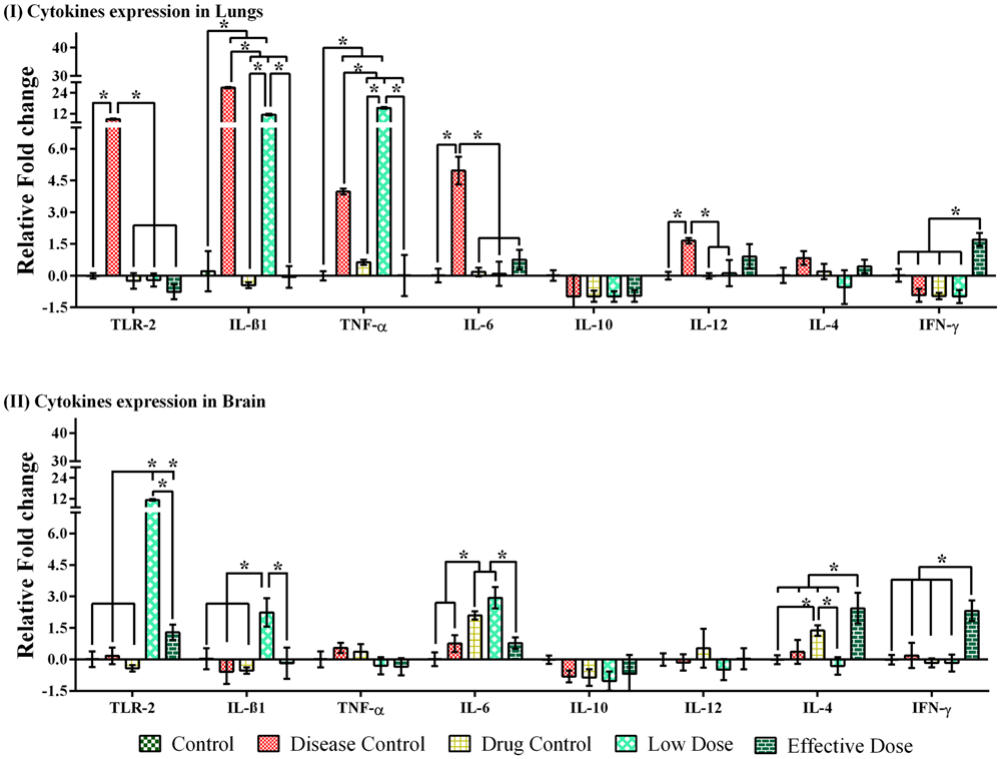
Cytokine gene expression in lung and brain in various study groups during treatment period in BALB/c mice. After the treatment period and animals were euthanized on 21 day post infection and lungs and brain were collected. Homogenized tissues were examined for expression of proinflmmatory cytokines. **(I)** TLR-2 expression was induced 8.8-fold in infected mice and cytokine expression (IL-β1, TNF-α, IL-6) were increased above the levels than control expect for IFN-γ and IL-10. **(II)** TLR-2 expression are elevated in low dose treated group. The cytokine levels in brain tissues were observed to be very low in disease group than that observed with any other treatment group, except TNF-α. The compound-**6** treated group had significantly higher expression of IL-β1 (2.23-fold), IL-6 (2.44-fold), IL-4 (2.44-fold), IFN-γ (2.31-fold). Bar represents mean and SD for triplicates. *Represents p ≤ 0.05; comparison by two-way ANOVA followed by Tukey’s multiple comparisons test (Supplementary Table S7) and graphs were obtained using GraphPad Prism software.

We explored the significance of compound-**6** in modulating cytokine expression and TLR-2 in infected and treated mice by RT-PCR. We found that TLR-2 expression was induced 8.8- fold in infected mice when compared to control (Fig. 5(I)). The expression of TLR-2 in drug control and low dose of compound-**6** was similar to that of control group. Whereas, the effective dose down regulated the TLR-2 expression. Infected mice had high fungal load as well as higher GXM levels in lungs, hence the expression of TLR-2 was also high in infected mice. Thus the down regulation of TLR-2 in effective dose treated mice suggests an effective fungicidal activity of compound-**6**, due to which there is a decrease in TLR-2 expression.

In infected mice, the cytokine mRNA expression was observed to be increased above the levels of control, except for IFN-γ and IL-10. The order of expression of inflammatory cytokines IL-β1, TNF-α, and IL-6 are as follows, infected mice > low dose > control, effective dose and AmpB (Fig. 5(I)). These results clearly show the potency of compound-**6** (effective dose) to elevate the expression of proinflammatory cytokines. The expression of other cytokines IL-10, IL-12, IL-4 and IFN-γ was very minimal in infected group when compared to IL-β1, TNF-α, and IL-6. The expression of IL-10 was not detected in any groups, the reason behind the down regulation of IL-10 is at present not clear. Interestingly, the down regulation is favorable as IL-10 and Th2 response are associated with susceptibility to infection and disease enhancement^37^. In case of IL-12 and IL-4, there was a little variation in the expression across all the groups. IL-12 and IL-4 expression were slightly reduced in compound-**6** (effective dose) treated group than infected one. A similar response was also observed in drug control. Interestingly, IFN-γ was down regulated in all groups except effective dose, with an upregulation by 1.7 fold (Fig. 5(I)).

### Cytokine expression in brain

TLR-2 expression was very much elevated in compound-**6** (low dose) treated mice than any other groups, suggesting the ability of compound-**6** to induce TLR-2 expression and subsequent upregulation of IL-β1, IL-6, IL-4, IFN-γ and down regulation of TNF-α, IL-10, IL-12.

The cytokine levels in brain were observed to be minimal in disease group when compared with any other treatment groups, except TNF-α. The compound-**6** treated group had significantly higher expression of IL-β1 (2.23-fold), IL-6 (2.44-fold), IL-4 (2.44-fold) IFN-γ (2.31-fold), suggesting the immunomodulatory property of compound-**6** against *C. neoformans* (Fig. 5(II)).

## Discussion

The pathology of cryptococcal infection is quite complex with host-pathogen interaction and host immune responses determining disease progress and therapeutic outcome. Development of resistance and adverse side effects are the two major issues with current line of treatment. Thus, it creates high demand to discover and promote new antifungal compounds that are effective and less toxic. This fact lead us to continue with our previous work^35^, to synthesis furanone derivatives with 5 positions dihydro-3,3-dimethyl-2(3 H)-furanone, during which two furanone compounds were synthesized (compound**-6** and compound**-11**). However, the antifungal activity was observed for compound-**6** (**(** *E*)-5-benzylidenedihydrofuran-2(3 *H*)-one) alone. The basic structural difference between compound-**6** and **11** is that compound-**11** has a phenyl-alleyen substituent. Due to the presence of two phenyl substitution, the compound-**11** has poor solubility in DMSO-H_2_O system. Probably this could be the reason for the lack of antifungal activity by compound-**11**.

From the results (Table 1 and Supplementary Fig. S1), it is clear that compound-**6** has excellent antifungal activities against the 7 tested strains. The decrease order of MIC_0.5_ values are found to be H99 > clinical strain > RPC29 and RPC30 (non-melanin strains) > BAC21 (acapsular strain) and ATCC 14116 (environmental strain) > CDC103 (acapsular strain), suggesting the significance of its major virulence factors (capsule and melanin) that resists the action of drugs. A similar kind of susceptibility pattern was observed for AmpB.

Though MIC of compound-**6** was found to be higher than AmpB (37 fold), the cell line toxicity study revealed the non-toxic nature of compound-**6** in HepG2 and J774.A.1 cell lines, with remarkable observation of 100% viability of immune cell lines. J774.A.1 macrophage cell lines were used to check the *in vitro* immunomodulation property of compound-**6** as they have a predisposition to Th2 response and we wanted to check whether there is a significant immunomodulation of macrophages by compound-**6**. Macrophages treated with compound-**6** were compared with LPS treated and untreated macrophages. LPS treated macrophages have been shown to produce both proinflammatory and anti-inflammatory cytokine responses^38,39^. Enhanced TLR-2 and proinflammatory cytokine gene expression of macrophages suggests the potency of compound-**6** to activate the TLR-2 expression and subsequently enhance the inflammatory cytokines (IL-β1, TNF-α, IFN-γ, IL-12). At the same time, compound-**6** had an inhibitory effect on anti-inflammatory cytokines (IL-4 andIL10). Hence by promoting TLR-2 linked proinflammatory responses and suppressing anti-inflammatory responses, compound**-6** was able to produce immunomodulation that could effectively contribute to pathogen killing. Though, as expected, LPS-induced inflammatory cytokine gene expression was comparatively higher than the compound**-6** treated macrophages, we are tempted to speculate that the ability of compound**-6** to stimulate only proinflammatory cytokines even in the absence of antigen is a significant property of the compound which could contribute to effective recognition and killing of the pathogen, viz., *C. neoformans*.

Based on the *in vitro* activity, we were curious to analyse the toxicity and therapeutic efficacy in a mice model. The drug induced toxicity assessment in BALB/c mice was particularly encouraging since compound-**6** was proved to be non-toxic even at a relatively higher concentration (500XMIC). The animal behavior, body weight, feed intake and gross observation of systemic organs were comparable to that of control group, suggesting the absence of compound-**6** induced adverse side effects. (Fig. 3(III)). We then investigated the therapeutic efficacy of compound-**6** in pulmonary cryptococosis model using *C. neoformans* H99 strain, which is well known for its hypervirulence in mice model^40,41^. Parallely, the efficacy of compound-**6** to reach target organs in disseminated infections was also studied, because most antifungal drugs exert their effects on microorganisms residing within tissues^42^. Understanding the penetration of drug into the site of infection is a key requirement to eliminate the pathogen^43^, hence we analysed the bioavailability of compound**-6** in liver, lung, kidney and brain, and this was correlated with reduction of fungal colony forming units (CFU) in all organs. Interestingly, compound-**6** was found to be distributed in all major organs with highest concentration in the target organ (lungs), followed by kidney, brain and liver. The bioavailability and reduction in yeast proliferation can be highly correlated with mouse survival. These notable results confirmed the ability of compound-**6** to cross the blood brain barrier. The low molecular weight (174 Da) and the presence of aromatic ring in compound-**6** favors the high degree to cross blood brain barrier^44^. The therapeutic efficacy of compound-**6** was concurrent with lung histopathology results, which was marked with increased clearance of the yeast from the lungs and decreased lung pathology (Fig. 4(I)). Taken together the antifungal activity, biodistribution and mice survival, confirm the promising activity of compound-**6** against H99 infection.

Both innate and adaptive defense responses are involved against *C. neoformans* infections^45^. Furthermore, the Th1 response is suggested to confer better clinical prognosis when compared to Th2. The higher levels of Th1 cytokines, TNF-α and IFN-γ are associated with increased fungal clearance^46,47^. Th2 cytokines such as IL-4 and IL-10 have been shown to have non-protective response against cryptococcal infection and are associated with poor clinical improvement^48,49^. The status of immune response is crucial for establishment of infection and dissemination. Hence, understanding the interactions between pathogens, drugs and immune responses are crucial to combat CM. In conjunction, we extended our investigation, to analyze the influence of compound-**6** on TLR-2 and cytokine expression in all 5 groups. TLR-2 have been recognized as an important pathogen recognition receptors and modulating cytokine production^50,51^. In infected mice, lung TLR-2 expression was high in contrast to brain. This was in concurrent with a previous report which demonstrated that TLR-2 expression was significantly reduced in CM patients^52,53^. In terms of cytokine expression, compound-**6** treated mice had significantly higher expression of IL-6 (lung and brain), IL-4 (lung and brain), IFN-γ (lung and brain), TNF-α, IL-β1and IL-12 (lung) when compared to control group (without infection). Similarly, when comparing compound-**6** treated and untreated group (disease control); compound-**6** has shown to increase the expression of IFN–γ and IL-4 in both lungs and brain. In addition to that, elevation of TLR-2 expression in brain was noticed. Collectively, the *in vitro* and *in vivo* cytokine expression demonstrated the higher expression of the prominent proinflammatory cytokines but not the anti-inflammatory cytokines suggesting the immunomodulatory property of compound-**6** (Fig. 5). Th1 cytokines IL-6, IL-12, TNF-α, IFN-γ expression are associated with protective response against *C. neoformans*, with significant outcomes in patients of HIV associated CM^54^. Interestingly, the expression of non-protective Th2 cytokine IL-10 was downregulated across all the groups, suggesting that compound**-6** induces the production of protective Th1 type cytokines. Although Th1 response are predominantly developed in CM patients and experimental model (H99 infected mice), dissemination of fungus to the brain and resulting death remains an unsolved problem. Hence antifungal agents with immunomodulatory potentials have gained attention in recent years.

**Figure 6:**
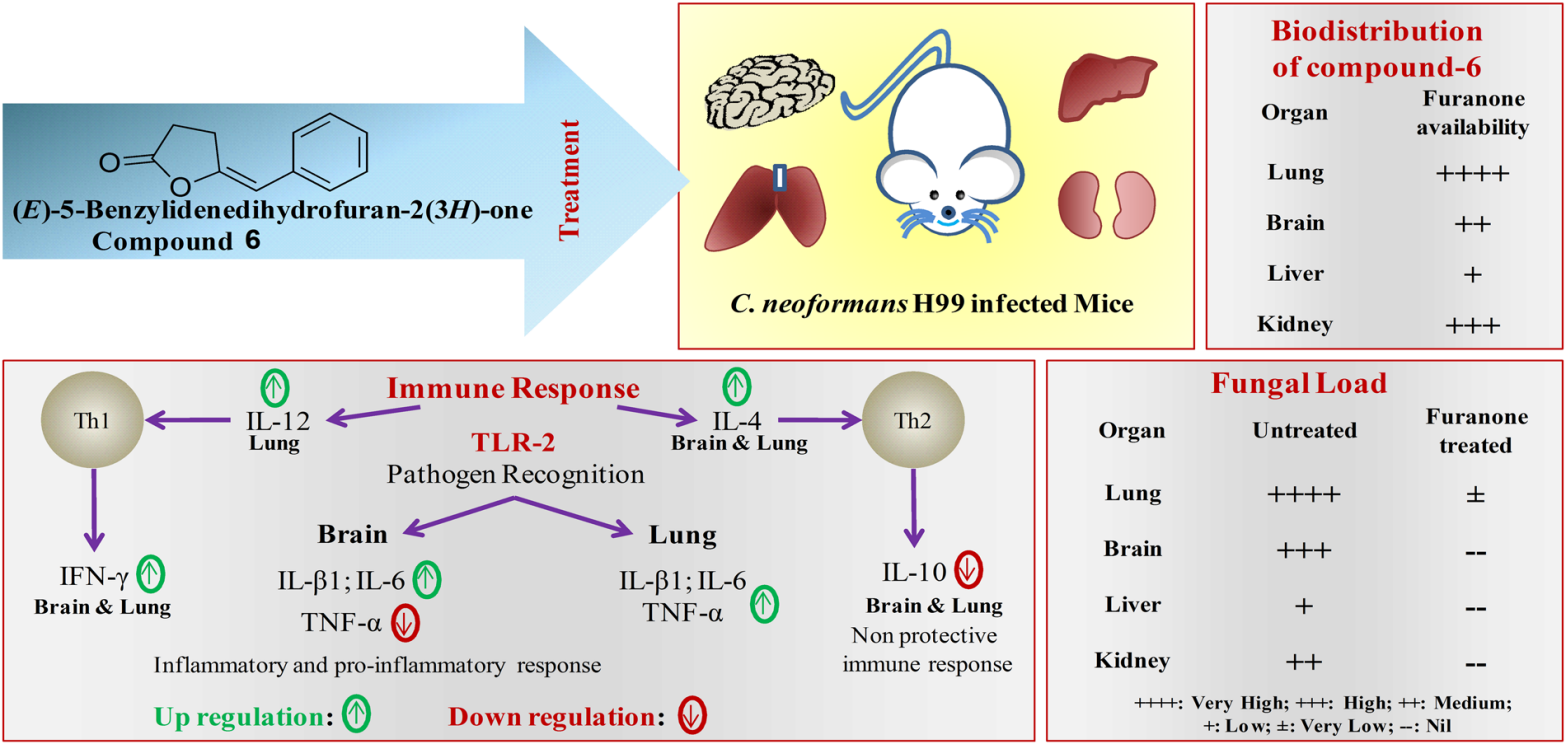
Therapeutic effect of compound-6. Integrated activities of antifungal and immunomodulation of compound-**6** to tackle *C. neoformans* H99 infection in mice model.

In summary, the results indicate that inhibition of yeast proliferation and thus mice survival is possibly due to the potential antifungal activity and improved host fungal clearance by compound**-6** in lungs and brain. Thus, the integrated activities of antifungal and immunomodulation of compound**-6** protect the mice from killing effect of H99 strain (Fig. 6). Until now, globally the potentiality of furanone compound to treat *C. neoformans* was not explored. The present study suggests the potential application of (*E*)-5-Benzylidenedihydrofuran-2(3 *H*)-one as an anticryptococcal and immunomodulatory compound to combat disseminated cryptococosis.

## Materials and Methods

### Strains

The details of *C. neoformans* strains used in the present study are provided in Table 2. The strains were maintained on PDA agar slants (4 °C) and 15% glycerol stocks at −80 °C.

**Table 2:** *C. neoformans* strains details.

Strain	Serotype	Genotype/Phenotype	Acknowledgment	Reference
H99	Serotype – A	Wild Type	Joseph Heitman (Duke University, Durham, North Carolina, USA)	65
BAC21	Serotype – D	MATα gpa1::ADE2 (G-protein gene deletion - Acaspular strain)		66
CDC103	Serotype – D	MATα pka1::ADE2 pka2∆::URA5 ura5 ade2 (Protein kinase A 1 and 2 gene deletion - Acapsular strain		
RPC29	Serotype – A	MATα ura5 lac1::URA5 lac2::neo (Laccase gene deletion - non-pigment producing strain)		65
RPC30	Serotype – A	MATα ura5 lac1::URA5 lac2::neo (Laccase gene deletion - non-pigment producing strain)		
ATCC 14116	Unknown	Wild type	Microbial Culture Collection Centre at Chandigarh, India.	62
Clinical strain	Unknown	Wild type	Government medical college, Trichy, Tamil Nadu, India	

### Compound synthesis

All reagents and solvents were purchased from commercial suppliers (Avra, Sigma, Alfa Aesar) and used without further purification. The reactions were monitored by thin-layer chromatography (TLC) using merck silica gel 60 F254 and visualized by UV detection or using *p*-anisaldehyde stain or DNP stain or molecular iodine. ^1^H NMR spectra were recorded in CDCl_3_ at room temperature on a Bruker Advance 300 spectrometer operating at 300 MHz. Chemical shifts (δ) are expressed in ppm using tetramethylsilane (TMS) as internal standard and coupling constants (*J*) are given in Hz.

### Synthesis of (*E*)-5-benzylidenedihydrofuran-2(3 *H*)-one 6

#### Synthesis of diethyl 2-(3-phenylprop-2-yn-1-yl)malonate 3

**Figure 7:**
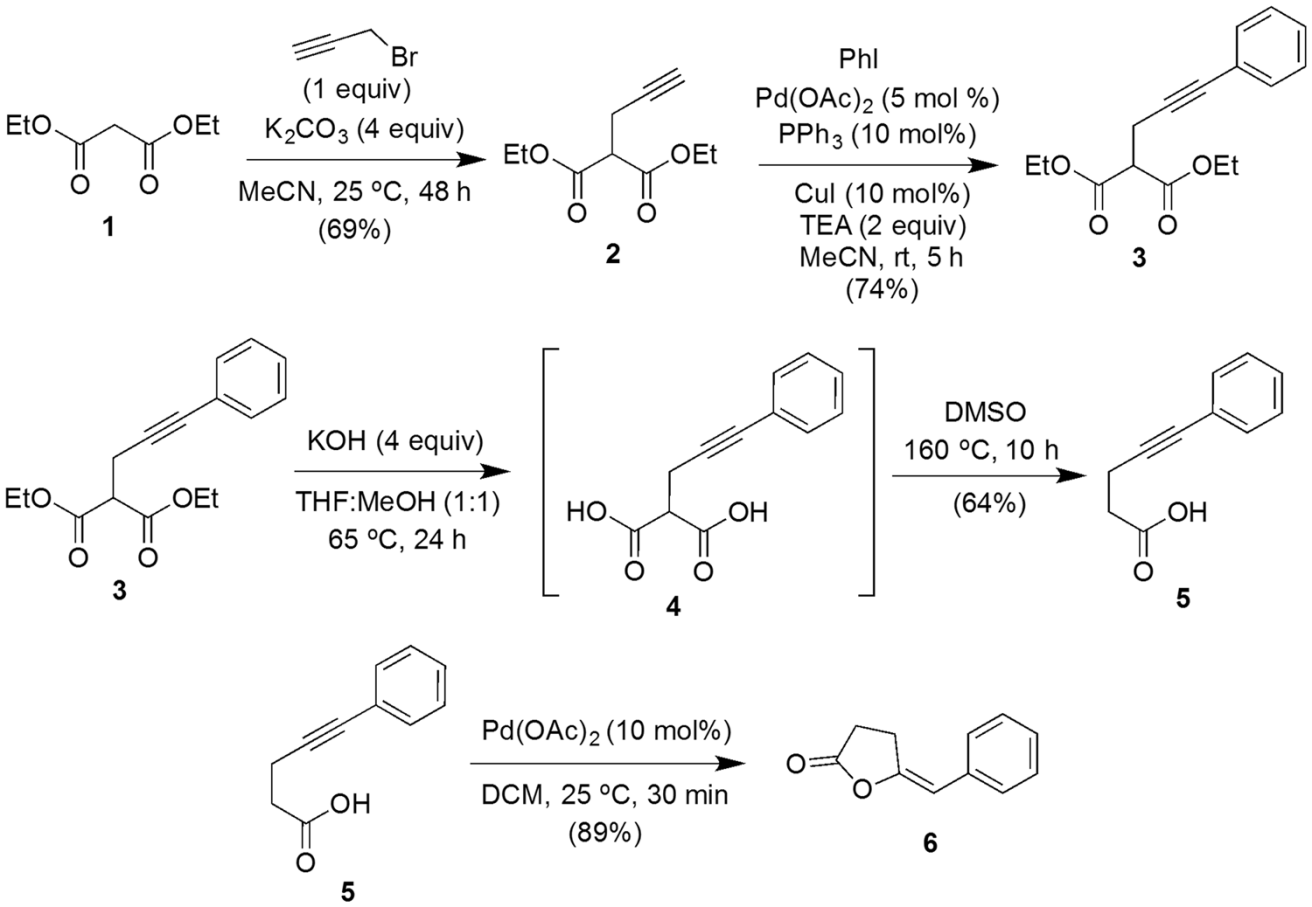
Scheme for the synthesis of (*E*)-5-Benzylidenedihydrofuran-2(3 *H*)-one (Compound-6).

To a stirred solution^55,56^ of diethyl malonate **1** (30 mmol) in acetonitrile (80 mL) were added K_2_CO_3_ (120 mmol) and propargyl bromide (30 mmol) and stirring was continued for 48 hours at 25 °C. After completion of the reaction, as indicated by TLC, the reaction mixture was diluted with water and extracted with dichloromethane (2 × 100 mL). The combined organic layer was washed with water (1 × 50 mL) followed by brine (1 × 50 mL), dried over anhydrous Na_2_SO_4_ and concentrated under reduced pressure. The residue was purified by silica gel column chromatography using petroleum ether-ethyl acetate mixture as eluent (95:5 v/v) to obtained compound-**2** (Fig. 7).

To a solution of diethyl 2-(prop-2-yn-1-yl)malonate **2** (20 mmol) and iodobenzene (20 mmol) in acetonitrile (60 mL) were added Pd(OAc)_2_ (5 mol%), PPh_3_ (10 mol%), CuI (10 mol%) and Et_3_N (40 mmol). The resulting mixture was degassed with nitrogen and stirred at 25 °C for 5 hours. After completion of the reaction, as indicated by thin layer chromatography (TLC), the reaction mixture was filtered through a pad of celite and washed well with EtOAc (2 × 50 mL). The organic layer was washed with water, brine and dried over anhydrous Na_2_SO_4_. The solvent was evaporated to dryness under reduced pressure and the crude mixture was chromatographed over silica using petroleum ether and ethyl acetate (EtOAc) (95:5, v/v) as eluent to obtain compound-**3** (Fig. 7).

### Synthesis of 5-phenylpent-4-ynoic acid 5

Compound-**3** (10 mmol) was dissolved in a 1:1 mixture of THF/MeOH (40 mL) and KOH (40 mmol) in H_2_O (4 mL) was added and the mixture was refluxed for 24 hours. After cooling, the reaction mixture was diluted with water and acidified with 2 N HCl, extracted with dichloromethane, washed with water, brine and dried over anhydrous Na_2_SO_4_. The solvent was evaporated and DMSO (25 mL) was added to the crude mixture and heated at 160 °C with stirring for 10 hours. The mixture was cooled to room temperature, diluted with water and extracted with dichloromethane. The organic layer was washed with brine, dried over anhydrous Na_2_SO_4_ and the solvent was evaporated. The crude mixture was purified through silica column chromatography using hexane/EtOAc (90:10, v/v) as eluent to obtain compound-**5** (Fig. 7).

### Synthesis of (*E*)-5-benzylidenedihydrofuran-2(3 *H*)-one 6

To a stirred solution of alkynoic acid **5** (5 mmol) in DCM (20 mL) at 25 °C under N_2_ was added Pd(OAc)_2_ (10 mol%) and stirring was continued. After complete consumption of the starting material, as indicated by TLC, the reaction mixture was filtered through a short pad of silica gel and the solvent was evaporated. The residue was purified through silica column chromatography using hexane/EtOAc (95:5 v/v) as an eluent to get the desired product **6 (**Fig. 7 **)**.

#### (E)-5-Benzylidenedihydrofuran-2(3 H)-one 6

Colourless solid; yield: 89%; ^1^H NMR (300 MHz, CDCl_3_): δ 2.69-2.75 (m, 2 H), 3.04 (td, *J* = 6.6, 1.5 Hz, 2 H), 5.55 (s, 1 H), 7.21 (td, *J* = 7.2, 1.2 Hz, 1 H), 7.33 (t, *J* = 7.8 Hz, 2 H), 7.55 (dd, *J* = 7.2, 1.2 Hz, 2 H).

### Synthesis of (E)-5-benzylidene-3-(3-phenylprop-2-yn-1-yl)dihydrofuran-2(3 H)-one 11 Synthesis of diethyl 2,2-bis(3-phenylprop-2-yn-1-yl)malonate 8

**Figure 8:**
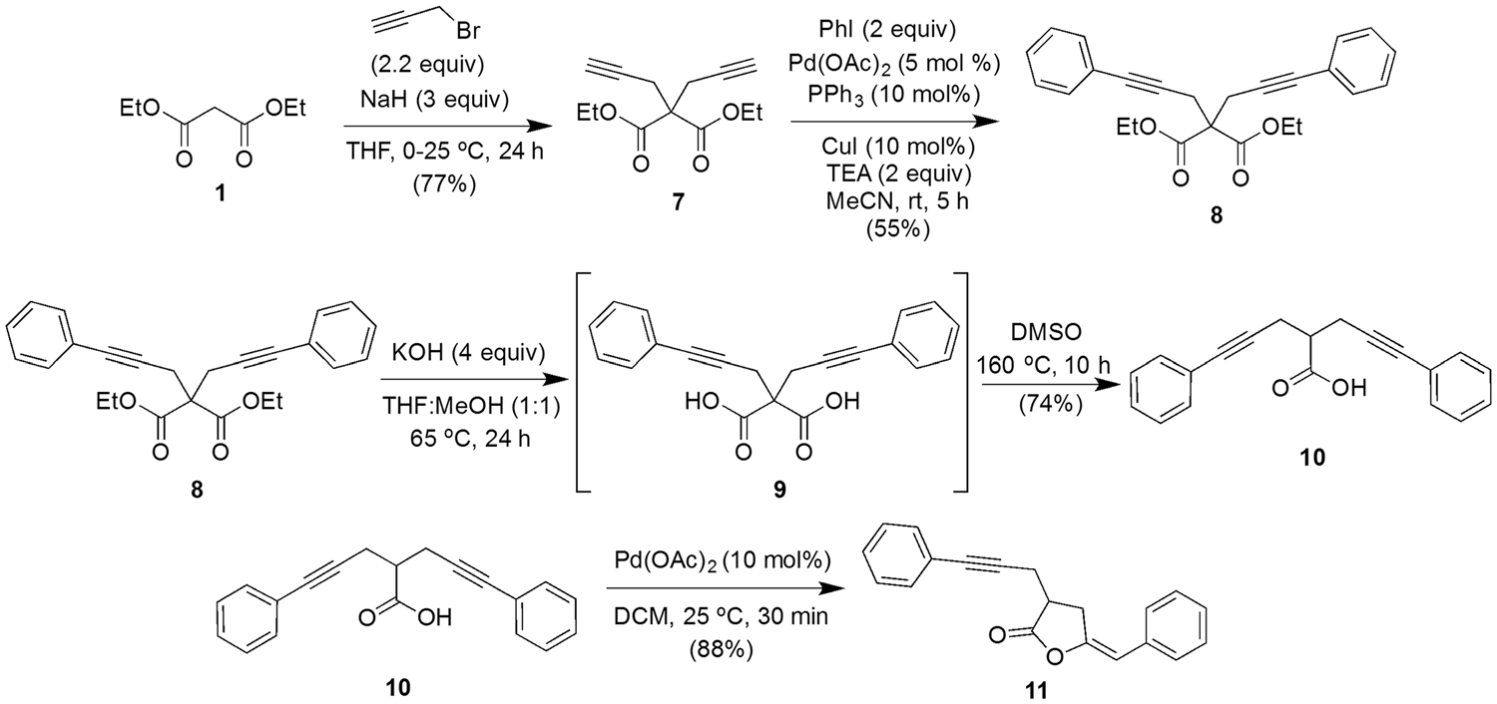
Scheme for the synthesis of (*E*)-5-Benzylidene-3-(3-phenylprop-2-ynyl)dihydrofuran-2(3 *H*)-one (compound-11).

To a stirred suspension^56^ of NaH (9 mmol) in THF (20 mL) at 0 °C under nitrogen atmosphere was added diethyl malonate **1** (3 mmol) dropwise and the mixture was stirred for additional 1 hour at room temperature. The reaction mixture was again cooled to 0 °C, propargyl bromide (6.6 mmol) was added dropwise and stirring was continued at 25 °C for 24 hours. The reaction was quenched with saturated NH_4_Cl solution, extracted with dichloromethane, washed with brine, dried over anhydrous Na_2_SO_4_ and evaporated. The crude product was purified through silica column chromatography using petroleum ether/EtOAc mixture (95:5, v/v) as eluent to furnish compound-**7 (**Fig. 8 **)**.

Compound-**7** was converted into **8** following the procedure mentioned for the synthesis of compound-**3** (2 equivalents of iodobenzene was used).

### Synthesis of 5-phenyl-2-(3-phenylprop-2-yn-1-yl)pent-4-ynoic acid 10

Compound-**10** was synthesized from compound-**8** following the procedure given for the compound-**5** synthesis from compound-**3** (Fig. 8).

### (*E*)-5-Benzylidene-3-(3-phenylprop-2-ynyl)dihydrofuran-2(3 *H*)-one 11

Compound-**11** was synthesized from compound-**10** following the procedure given for the compound-**6** synthesis from compound-**5 (**Fig. 8).

#### (E)-5-Benzylidene-3-(3-phenylprop-2-ynyl)dihydrofuran-2(3 H)-on**e 11**

Yellow solid; Yield: 88%; ^1^H-NMR (300 MHz, CDCl_3_): δ = 2.89–2.98 (m, 2 H), 3.07–3.27 (m, 3 H), 5.61 (s, 1 H), 7.18–7.29 (m, 4 H), 7.30–7.39 (m, 4 H), 7.54–7.57 (m, 2 H).

### Minimal inhibitory concentration determination (MIC)

MIC of compound**-6** and compound-**11** against 7 different strains of *C. neoformans* were determined by microbroth dilution assay in accordance with CLSI protocols using RPMI 1640 medium with MOPS^57–59^ was used. The activity was also checked for a clinical strain of *Candida albicans*, another significant opportunistic yeast pathogen. AmpB was used as drug control. The MICs were recorded as the concentration at which 90% turbidity was reduced, relative to the turbidity of control. Compound**-6**, **11** and AmpB were dissolved in 1% DMSO. Various concentrations of compound**-6** and **11** (100 to 600 μg/mL) and AmpB (0-16 μg/mL) were tested. Each well contained 100 µl of media with 10^6^ CFU/mL and varying concentrations of compound**-6**, **11** and AmpB. The turbidity was measured at 620 nm after 48 hours incubation followed by viability assay by spot plate method on PDA plates. Duplicates were maintained for each concentration.

### Cytotoxicity assay of compound-6

Further studies were carried with compound**-6** alone, as compound-**11** does not show activity against *C. neoformans*. The *in vitro* cytotoxicity assay was performed in HepG2 human hepatocyte cell line and J774.A.1 macrophage cell line procured from National Center for Cell Science, Pune, India. The MTT toxicity assay was performed between 100 to 600 μg/mL in controlled conditions (5% CO_2,_ 95% humidity, 37 °C) at 12 hours time intervals for 24 hours. Triplicates were maintained and the experiment was repeated twice. The differences were tested for statistical significance by student’s t test.

### *In vitro* immunomodulation of compound-6

The role of compound-**6** in modulating cytokine gene expression was analysed using J774.A.1 macrophage cell line. The cells were maintained in Dulbecco’s Modified Eagle’s Medium (DMEM: AL067A, Himedia) with 10% Fetal Bovine Serum (FBS: RM9955, Himedia) and 1X antibiotics (A004, Himedia). The macrophage cells were serum starved and used for further studies.

Transcription pattern of cytokines expression were analyzed for (i) cells treated with lipopolysaccharide (LPS: L9143, Sigma) (3 μg/mL) with interferon gamma (IFN-γ: PHP050, BIO-RAD) (100 pmol) (ii) cells treated with compound-**6** (iii) untreated cells. The cells mentioned in the above conditions were maintained in DMEM with 10% FBS without antibiotics for 24 hours. After the treatment period, the cells were washed with serum free medium and total RNA (2–10 μg) were extracted using TRIzol method (30006, Takara) and samples were converted into cDNA by PrimeScript™ RT-PCR Kit (RR014A, Clontech, Takara) and tested in triplicate on a Real-Time PCR System (Eppendorf AG22331, Germany) using DyNAmo Flash STBR Green qPCR kit (416 L, Thermo Scientific). The following cytokines genes were amplified by initial denaturation at 95 °C for 5 minutes followed by 40 cycles at 95 °C for 30 seconds, annealing at 61 °C (GAPDH, IL-12), 62 °C (IL-10), 65 °C (TLR-2), 60 °C (IL-β1), 57 °C (IL-4), 59 °C (IL-6), 58 °C (TNF-α) and 55 °C (IFN-γ) for 30 seconds and extension at 72 °C for 30 seconds and analyzed with the 2^−∆∆Ct^ method and normalized with GAPDH, and control. Primer sequence for PCR is listed in Table 3.

**Table 3:** List of primers used for cytokine expression.

Gene		Sequence	Product size	Cycles	Reference
**TLR-2**	Forward	5′-TCGTTGTTCCCTGTGTTGCT-3′	**389**	**30**	67
	Reverse	5′-CCACGCCCACATCATTCTCA-3′			
**IL-β1**	Forward	5′-ATGGCAACTGTTCCTGAACTCAACT-3′	**563**	**35**	68,69
	Reverse	5′-CAGGACAGGTATAGATTCTTTCCTTT-3′			
**TNF-α**	Forward	5′-GAGCTTTCAACAACTACTCAG-3′	**276**	**30**	70
	Reverse	5′-GGAAGGCCTGAGATCTTATC-3′			
**IL-6**	Forward	5′-GCCTATTGAAAATTTCCTCTG-3′	**310**	**35**	71
	Reverse	5′-TAGGTTTGCCGAGTAGATCTC-3′			
**IL-10**	Forward	5′-CGGGAAGACAATAACTG-3′	**186**	**35**	70
	Reverse	5′-CATTTCCGATAAGGCTTGG-3′			
**IL-12**	Forward	5′-CGT GCT CAT GGC TGG TGC AAA G-3′	**220**	**30**	70
	Reverse	5′-CTT CAT CTG CAA GTT CTT GGG C-3′			
**IL-4**	Forward	5′-TCG GCA TTT TGA ACG AGG TC-3′	**216**	**30**	70
	Reverse	5′-GAA AAG CCC GAA AGA GTC TC-3′			
**IFN-γ**	Forward	5′-GGTTGGACAAAAAGAATCTG-3′	**227**	**35**	72
	Reverse	5′-ACCACAGAGAGCAAGGACTT-3′			
**GAPDH**	Forward	5′-CTGCCCAGAACATCATCCCT-3′	**266**	**35**	73
	Reverse	5′-GGTCCTCAGTGTAGCCCAAGA-3′			

### Ethics

Animal experiments were performed according to the guidelines of animal experimentation of Central Animal Facility (CAF), National Accreditation Board for Testing and Calibration Laboratories (NABL) accredited (ISO-IEC 17025), SASTRA University. The experimental protocol was approved by Institutional Animal Ethics Committee (IAEC), SASTRA University, Thanjavur, Tamil Nadu, India (IAEC approval number: 427/SASTRA/IAEC/RPP).

### Acute toxicity study

Male BALB/c mice (5–6 weeks old, 29.33 ± 2.1 g) were used for acute toxicity and efficacy studies (purchased from CAF, SASTRA University). The animals were housed in sterile and controlled atmospheric conditions (24 ± 0.5 °C, and 65 ± 5% humidity). Fixed dose procedure was followed to determine acute toxicity of compound**-6** ^60^. A group of six mice were intravenously injected with 500-fold higher MIC of compound**-6** (200 mg/mL) dissolved in 1% DMSO as a single dose. The maximum concentration of compound**-6** (200 mg/kg) was limited due to the toxicity of vehicle at more than 1%. Mice survival, body weight, feed intake and behavioral change were observed for 14 days.

### Efficacy determination

Immunosuppression were induced in 5 groups (n = 6) of male BALB/c mice (5–6 weeks old, 32.5 ± 3.3 g) by intraperitoneal injection of 10 mg/kg/day dexamethasone (Dexalab, Laborate Pharmaceuticals India LTD., India) till the day of sacrifice^61^. *C. neoformans* H99 strain was used for *in vivo* study. The strain was inoculated in potato dextrose broth supplemented with 20 mM MgSO_4_.7H_2_O to increase capsule production^62^. Mice of disease and treatment groups were infected with 10^4^ cell/24 µl of *C. neoformans* H99 strain through intratracheal route^63^. The antifungal treatment was started at 48 hours post-infection (hpi). Single dose of Compound**-6** at two concentrations (20 mg/kg/day and 100 mg/kg/day) were administered intravenously. The drug control group received AmpB (0.5 mg/kg/day)^64^. After the treatment period, mice from all groups were euthanized by CO_2_ inhalation on 21 day post infection and organs (brain, lung, kidney, liver) were collected to determine fungal burden, biodistribution of drug, cytokine expression, and histopathology. Statistical analysis was done for analysis.

### Survival studies

The efficacy of compound**-6** was confirmed by observing the survival of mice. During the treatment period, body weight, and feed intake were recorded daily. For histopathological examination, the organs were fixed in 10% neutral buffered formalin. Sections were prepared and stained with haematoxylin and eosin.

### Determination of fungal burden

100 mg of each organ was suspended in 1 mL of sterile phosphate buffer saline (PBS) followed by homogenization aseptically with micro pestles and were 10-fold diluted in sterile PBS. An aliquot of homogenate was plated on PDA plates and incubated for 72 hours at 37 °C. Yeast colonies were counted and the fungal burden in each organ was calculated in term of CFU/mg.

### Biodistribution of Drug

Homogenised sample (in 1 × PBS) of 100 mg of each organ were centrifuged at 1500 g for 10 minutes and resulting supernatants were mixed with EtOAc. The EtOAc extract was dried at room temperature and dissolved in 0.2 µl methanol (HPLC grade). Samples were then filtered through a 0.2 µm pore size membrane syringe filter and analyzed in reverse phase HPLC (Agilent 1260-Infinity, C-18 column 4.7 × 250 mm; 5 µm particle size) at 25 °C. The mobile phase consisted of 0.005 M EDTA buffer, acetonitrile and methanol (50:49:1 v/v) at 1 mL/minute flow rate. The concentrations of compound**-6** and AmpB were quantified by comparing the peak area, retention time (RT) and wavelength with a standard concentrations (0.25, 0.5 and 1 mg/mL). The RT of compound**-6** between 8.2 to 8.8 minutes at 200 nm and AmpB between 3.1 to 3.8 minutes at 400 nm were noted. The concentration of compound**-6** and AmpB in each organ was expressed in µg/g.

### Cytokine expression

Transcriptional pattern of cytokines in collected organs (lungs and brain) from all groups of animals were analysed. Tissue (100 mg) were washed with DEPC treated water and homogenized. Total RNA was extracted and converted into cDNA. The cytokines TLR-2, IL-β1, TNF-α, IL-6, IL-10, IL-12, IL-4 and IFN-γ genes were amplified to analyse the transcriptional expression in lungs and brain of all groups. Samples were tested in triplicate on a Real-Time PCR System (Eppendorf AG22331, Germany) and the amplification methods were followed same described for *in vitro* cytokine gene expression.

### Statistical analysis

All experiments were performed in duplicates or triplicates and the data analysis was executed in the GraphPad Prism 6. One-way and two-way ANOVA followed by post hoc test (Tukey’s multiple comparison) were performed to test statistical significance for multiple comparisons. All graphs were prepared with GraphPad Prism 6, and were expressed as the mean ± Standard deviation (SD) of duplicates or triplicates.

## Electronic supplementary material

Supplementary Files
