# Supplementary material for: Exploration of Antifungal and Immunomodulatory Potentials of a Furanone Derivative to Rescue Disseminated Cryptococosis in Mice
Source: Sci Rep. 2017 Nov 13;7:15400. doi: 10.1038/s41598-017-15500-8 (PMC5684196; doi:10.1038/s41598-017-15500-8)
Supplement: Supplementary file 1 — Supplementary Files [file 41598_2017_15500_MOESM1_ESM.pdf]

Exploration of Antifungal and Immunomodulatory Potentials of a Furanone Derivative to Rescue Disseminated Cryptococosis in Mice

Sudarshan Singh Rathore[1], Isravel Muthukrishnan[2], Sridharan Vellaisamy[2], C. Davidraj[3], Cheepurupalli Lalitha[1], Thiagarajan Raman[4], Jayapradha Ramakrishnan[1]*

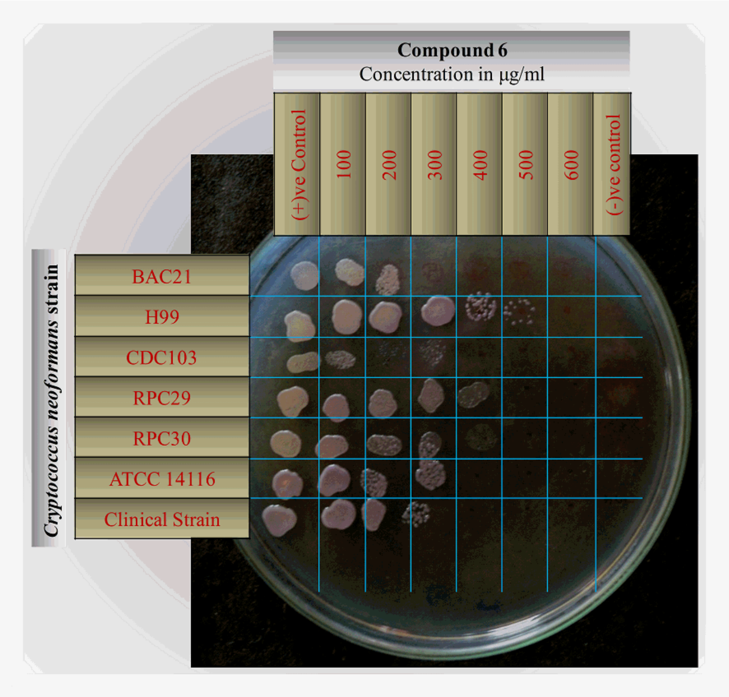

Supplementary Fig. S1: Spot plate method for compound-6 against 7 different strains of *C. neoformans*.

I. Cell line (J774.A.1) toxicity of Compound-6

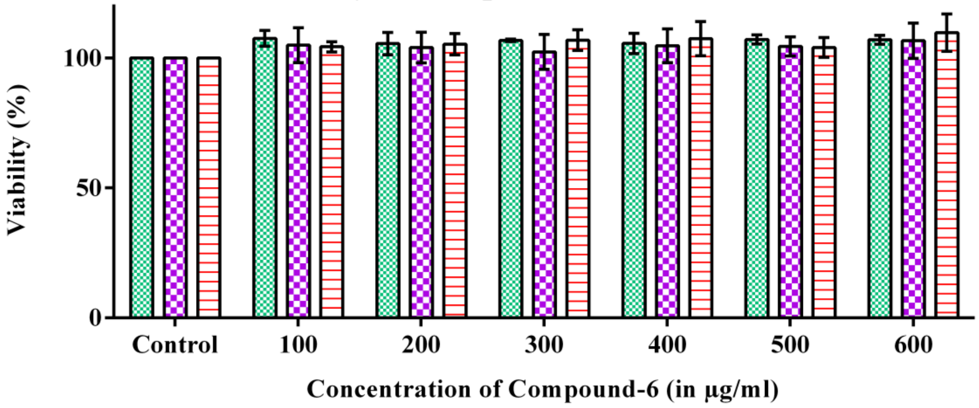

II. *In vitro* (J774.A.1 macrophage cell line) immunomodulation of Compound-6

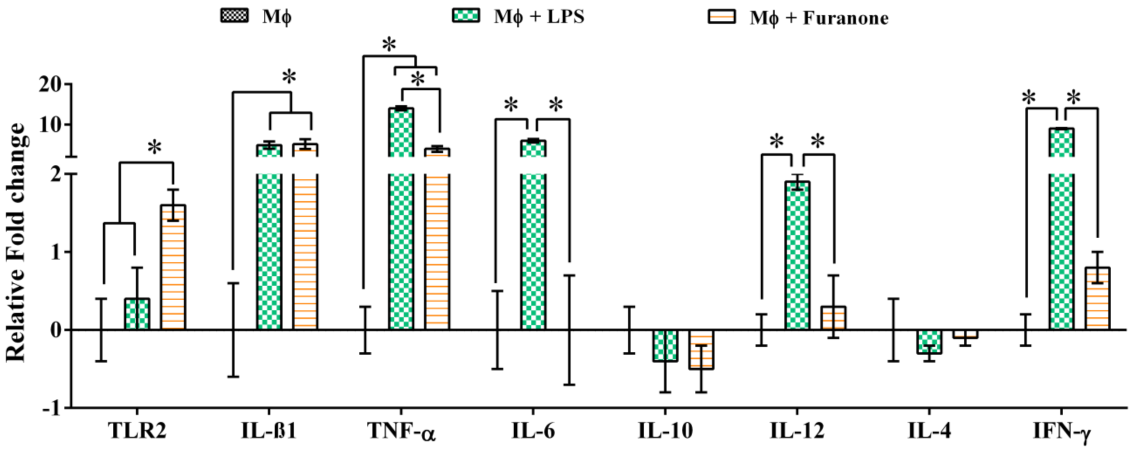

Supplementary Fig. S2: (I): *In vitro* cytotoxicity assay of compound-6 in J774.A.1 cell line. No toxicity was observed up to 600 µg/mL at 24 hours incubation respectively. **(II): *In vitro* immunomodulation study in J774.A.1 macrophage cell line.** The cytokine levels were observed for untreated, LPS + IFN-γ and compound-**6** treated macrophages. The compound-**6** treated cells had significantly higher expression of TLR-2 (1.6 fold), IL-β1 (5.2 fold), TNF-α (4.0 fold), IL-12 (0.3 fold), IFN-γ (0.8 fold) compared to untreated macrophage cells. Bar represents mean and SD for triplicates. * represents p≤0.05 from control and other variables respectively; comparison by two-way ANOVA followed by Tukey's multiple comparisons test (**Supplementary Table S2**) and graphs were obtained using GraphPad Prism software.

Supplementary Table S1: Two way ANOVA followed by Tukey's multiple comparisons test was performed for compound-**6** toxicity in HepG2 cell line.

**1.**	**HepG2 Cell line**					
	**Two-way ANOVA**	Ordinary				
	Alpha	0.05				
	**Source of Variation**	**% of total variation**	**P value**	**P value summary**	**Significant?**	
	Interaction	24.25	0.0006	***	Yes	
	Row Factor	46.46	< 0.0001	****	Yes	
	Column Factor	7.243	0.0521	Ns	No	
	**ANOVA table**	**SS**	**DF**	**MS**	**F (DFn, DFd)**	**P value**
	Interaction	1178	12	98.18	F (12, 42) = 3.851	P = 0.0006
	Row Factor	2257	2	1128	F (2, 42) = 44.26	P < 0.0001
	Column Factor	351.8	6	58.64	F (6, 42) = 2.300	P = 0.0521
	Residual	1071	42	25.49		
**1.1.**	**Tukey's multiple comparisons test**	**Mean Diff.**	**95% CI of diff.**	**Significant?**	**Summary**	
**1.1.1.**	**1hour**					
	0 vs. 100	-7.578	-20.34 to 5.184	No	ns	
	0 vs. 200	-1.582	-14.34 to 11.18	No	ns	
	0 vs. 300	-2.782	-15.54 to 9.980	No	ns	
	0 vs. 400	-3.631	-16.39 to 9.131	No	ns	
	0 vs. 500	-7.179	-19.94 to 5.583	No	ns	
	0 vs. 600	-7.079	-19.84 to 5.683	No	ns	
	100 vs. 200	5.996	-6.766 to 18.76	No	ns	
	100 vs. 300	4.797	-7.965 to 17.56	No	ns	
	100 vs. 400	3.947	-8.815 to 16.71	No	ns	
	100 vs. 500	0.3998	-12.36 to 13.16	No	ns	
	100 vs. 600	0.4997	-12.26 to 13.26	No	ns	
	200 vs. 300	-1.199	-13.96 to 11.56	No	ns	
	200 vs. 400	-2.049	-14.81 to 10.71	No	ns	
	200 vs. 500	-5.596	-18.36 to 7.166	No	ns	
	200 vs. 600	-5.496	-18.26 to 7.266	No	ns	
	300 vs. 400	-0.8494	-13.61 to 11.91	No	ns	
	300 vs. 500	-4.397	-17.16 to 8.365	No	ns	
	300 vs. 600	-4.297	-17.06 to 8.465	No	ns	
	400 vs. 500	-3.548	-16.31 to 9.214	No	ns	
	400 vs. 600	-3.448	-16.21 to 9.314	No	ns	
	500 vs. 600	0.0999	-12.66 to 12.86	No	ns	
**1.1.2.**	**12 hours**					

	0 vs. 100	-11.23	-23.99 to 1.536	No	ns	
	0 vs. 200	-11.43	-24.19 to 1.336	No	ns	
	0 vs. 300	-12.92	-25.69 to -0.1627	Yes	*	
	0 vs. 400	-12.38	-25.14 to 0.3869	No	ns	
	0 vs. 500	-13.12	-25.89 to -0.3626	Yes	*	
	0 vs. 600	-10.68	-23.44 to 2.086	No	ns	
	100 vs. 200	-0.1999	-12.96 to 12.56	No	ns	
	100 vs. 300	-1.699	-14.46 to 11.06	No	ns	
	100 vs. 400	-1.149	-13.91 to 11.61	No	ns	
	100 vs. 500	-1.899	-14.66 to 10.86	No	ns	
	100 vs. 600	0.5496	-12.21 to 13.31	No	ns	
	200 vs. 300	-1.499	-14.26 to 11.26	No	ns	
	200 vs. 400	-0.9494	-13.71 to 11.81	No	ns	
	200 vs. 500	-1.699	-14.46 to 11.06	No	ns	
	200 vs. 600	0.7495	-12.01 to 13.51	No	ns	
	300 vs. 400	0.5496	-12.21 to 13.31	No	ns	
	300 vs. 500	-0.1999	-12.96 to 12.56	No	ns	
	300 vs. 600	2.249	-10.51 to 15.01	No	ns	
	400 vs. 500	-0.7495	-13.51 to 12.01	No	ns	
	400 vs. 600	1.699	-11.06 to 14.46	No	ns	
	500 vs. 600	2.448	-10.31 to 15.21	No	ns	
**1.1.3.**	**24 hours**					
	0 vs. 100	2.415	-10.35 to 15.18	No	ns	
	0 vs. 200	0.4164	-12.35 to 13.18	No	ns	
	0 vs. 300	-2.931	-15.69 to 9.831	No	ns	
	0 vs. 400	1.965	-10.80 to 14.73	No	ns	
	0 vs. 500	10.06	-2.702 to 22.82	No	ns	
	0 vs. 600	18.41	5.647 to 31.17	Yes	**	
	100 vs. 200	-1.999	-14.76 to 10.76	No	ns	
	100 vs. 300	-5.346	-18.11 to 7.416	No	ns	
	100 vs. 400	-0.4497	-13.21 to 12.31	No	ns	
	100 vs. 500	7.645	-5.117 to 20.41	No	ns	
	100 vs. 600	15.99	3.232 to 28.76	Yes	**	
	200 vs. 300	-3.348	-16.11 to 9.414	No	ns	
	200 vs. 400	1.549	-11.21 to 14.31	No	ns	
	200 vs. 500	9.644	-3.118 to 22.41	No	ns	
	200 vs. 600	17.99	5.231 to 30.76	Yes	**	
	300 vs. 400	4.897	-7.865 to 17.66	No	ns	
	300 vs. 500	12.99	0.2294 to 25.75	Yes	*	
	300 vs. 600	21.34	8.579 to 34.10	Yes	***	
	400 vs. 500	8.095	-4.667 to 20.86	No	ns	
	400 vs. 600	16.44	3.682 to 29.21	Yes	**	

	500 vs. 600	8.349	-4.413 to 21.11	No	ns	
	**Tukey's multiple comparisons test**	**Mean Diffw**	**95% CI of diffw**	**Significant?**	**Summary**	
**1.1.4.**	**Control**					
	1h vs. 12h	0	-10w02 to 10w02	No	ns	
	1h vs. 24h	0	-10w02 to 10w02	No	ns	
	12h vs. 24h	0	-10w02 to 10w02	No	ns	
**1.1.5.**	**100 µg/ml**					
	1h vs. 12h	-3.647	-13w66 to 6w369	No	ns	
	1h vs. 24h	9.993	-0w02271 to 20w01	No	ns	
	12h vs. 24h	13.64	3w625 to 23w66	Yes	**	
**1.1.6.**	**200 µg/ml**					
	1h vs. 12h	-9.843	-19w86 to 0w1727	No	ns	
	1h vs. 24h	1.999	-8w017 to 12w01	No	ns	
	12h vs. 24h	11.84	1w826 to 21w86	Yes	*	
**1.1.7.**	**300 µg/ml**					
	1h vs. 12h	-10.14	-20w16 to -0w1271	Yes	*	
	1h vs. 24h	-0.1499	-10w17 to 9w866	No	ns	
	12h vs. 24h	9.993	-0w02278 to 20w01	No	ns	
**1.1.8.**	**400 µg/ml**					
	1h vs. 12h	-8.744	-18w76 to 1w272	No	ns	
	1h vs. 24h	5.596	-4w420 to 15w61	No	ns	
	12h vs. 24h	14.34	4w324 to 24w36	Yes	**	
**1.1.9.**	**500 µg/ml**					
	1h vs. 12h	-5.946	-15w96 to 4w070	No	ns	
	1h vs. 24h	17.24	7w222 to 27w25	Yes	***	
	12h vs. 24h	23.18	13w17 to 33w20	Yes	****	
**1.1.10.**	**600 µg/ml**					
	1h vs. 12h	-3.598	-13w61 to 6w418	No	ns	
	1h vs. 24h	25.49	15w47 to 35w50	Yes	****	
	12h vs. 24h	29.09	19w07 to 39w10	Yes	****	

Supplementary Table S2: Two way ANOVA followed by Tukey's multiple comparisons test was performed for compound-**6** immunomodulation in J774.A.1 cell line model.

1.	**J774.A.1 Cell line**					
	**Two-way ANOVA**	Ordinary				
	Alpha	0.05				
	**Source of Variation**	**% of total variation**	**P value**	**P value summary**	**Significant?**	
	Interaction	5.44	0.9888	ns	No	
	Row Factor	3.152	0.3806	ns	No	
	Column Factor	24.46	0.0335	*	Yes	
	ANOVA table	SS	DF	MS	F (DFn, DFd)	P value
	Interaction	68.73	12	5.727	F (12, 42) = 0w2844	P = 0w9888
	Row Factor	39.82	2	19.91	F (2, 42) = 0w9886	P = 0w3806
	Column Factor	309	6	51.51	F (6, 42) = 2w557	P = 0w0335
	Residual	845.9	42	20.14		
**1.1.**	**Tukey's multiple comparisons test**	**Mean Diffw**	**95% CI of diffw**	**Significant?**	**Summary**	
**1.1.1.**	**1h**					
	0 vs. 100	-7.578	-18w92 to 3w764	No	ns	
	0 vs. 200	-5.582	-16w92 to 5w760	No	ns	
	0 vs. 300	-6.782	-18w12 to 4w561	No	ns	
	0 vs. 400	-5.631	-16w97 to 5w712	No	ns	
	0 vs. 500	-7.179	-18w52 to 4w164	No	ns	
	0 vs. 600	-7.079	-18w42 to 4w264	No	ns	
	100 vs. 200	1.996	-9w347 to 13w34	No	ns	
	100 vs. 300	0.7968	-10w55 to 12w14	No	ns	
	100 vs. 400	1.947	-9w395 to 13w29	No	ns	
	100 vs. 500	0.3998	-10w94 to 11w74	No	ns	
	100 vs. 600	0.4997	-10w84 to 11w84	No	ns	
	200 vs. 300	-1.199	-12w54 to 10w14	No	ns	
	200 vs. 400	-0.0486	-11w39 to 11w29	No	ns	
	200 vs. 500	-1.596	-12w94 to 9w746	No	ns	
	200 vs. 600	-1.496	-12w84 to 9w846	No	ns	
	300 vs. 400	1.151	-10w19 to 12w49	No	ns	
	300 vs. 500	-0.397	-11w74 to 10w95	No	ns	
	300 vs. 600	-0.2971	-11w64 to 11w05	No	ns	
	400 vs. 500	-1.548	-12w89 to 9w795	No	ns	
	400 vs. 600	-1.448	-12w79 to 9w895	No	ns	
	500 vs. 600	0.0999	-11w24 to 11w44	No	ns	

**1.1.2.**	**12h**					
	0 vs. 100	-4.93	-16w27 to 6w413	No	ns	
	0 vs. 200	-4.031	-15w37 to 7w312	No	ns	
	0 vs. 300	-2.332	-13w67 to 9w011	No	ns	
	0 vs. 400	-4.73	-16w07 to 6w612	No	ns	
	0 vs. 500	-4.48	-15w82 to 6w862	No	ns	
	0 vs. 600	-6.629	-17w97 to 4w714	No	ns	
	100 vs. 200	0.8994	-10w44 to 12w24	No	ns	
	100 vs. 300	2.598	-8w744 to 13w94	No	ns	
	100 vs. 400	0.1998	-11w14 to 11w54	No	ns	
	100 vs. 500	0.4497	-10w89 to 11w79	No	ns	
	100 vs. 600	-1.699	-13w04 to 9w644	No	ns	
	200 vs. 300	1.699	-9w644 to 13w04	No	ns	
	200 vs. 400	-0.6996	-12w04 to 10w64	No	ns	
	200 vs. 500	-0.4497	-11w79 to 10w89	No	ns	
	200 vs. 600	-2.598	-13w94 to 8w744	No	ns	
	300 vs. 400	-2.398	-13w74 to 8w944	No	ns	
	300 vs. 500	-2.149	-13w49 to 9w194	No	ns	
	300 vs. 600	-4.297	-15w64 to 7w046	No	ns	
	400 vs. 500	0.2499	-11w09 to 11w59	No	ns	
	400 vs. 600	-1.899	-13w24 to 9w444	No	ns	
	500 vs. 600	-2.149	-13w49 to 9w194	No	ns	
**1.1.3.**	**24h**					
	0 vs. 100	-4.33	-15w67 to 7w012	No	ns	
	0 vs. 200	-5.33	-16w67 to 6w013	No	ns	
	0 vs. 300	-6.879	-18w22 to 4w464	No	ns	
	0 vs. 400	-7.478	-18w82 to 3w864	No	ns	
	0 vs. 500	-4.031	-15w37 to 7w312	No	ns	
	0 vs. 600	-9.727	-21w07 to 1w616	No	ns	
	100 vs. 200	-0.9994	-12w34 to 10w34	No	ns	
	100 vs. 300	-2.548	-13w89 to 8w794	No	ns	
	100 vs. 400	-3.148	-14w49 to 8w195	No	ns	
	100 vs. 500	0.2998	-11w04 to 11w64	No	ns	
	100 vs. 600	-5.396	-16w74 to 5w946	No	ns	
	200 vs. 300	-1.549	-12w89 to 9w794	No	ns	
	200 vs. 400	-2.149	-13w49 to 9w194	No	ns	
	200 vs. 500	1.299	-10w04 to 12w64	No	ns	
	200 vs. 600	-4.397	-15w74 to 6w946	No	ns	
	300 vs. 400	-0.5996	-11w94 to 10w74	No	ns	

	300 vs. 500	2.848	-8w495 to 14w19	No	ns	
	300 vs. 600	-2.848	-14w19 to 8w495	No	ns	
	400 vs. 500	3.448	-7w895 to 14w79	No	ns	
	400 vs. 600	-2.249	-13w59 to 9w094	No	ns	
	500 vs. 600	-5.696	-17w04 to 5w646	No	ns	
	**Tukey's multiple comparisons test**	**Mean Diffw**	**95% CI of diffw**	**Significant?**	**Summary**	
**1.1.4.**	**Control**					
	1h vs. 12h	0	-8w902 to 8w902	No	ns	
	1h vs. 24h	0	-8w902 to 8w902	No	ns	
	12h vs. 24h	0	-8w902 to 8w902	No	ns	
**1.1.5.**	**100 µg/ml**					
	1h vs. 12h	2.648	-6w254 to 11w55	No	ns	
	1h vs. 24h	3.248	-5w654 to 12w15	No	ns	
	12h vs. 24h	0.5996	-8w303 to 9w502	No	ns	
**1.1.6.**	**200 µg/ml**					
	1h vs. 12h	1.552	-7w350 to 10w45	No	ns	
	1h vs. 24h	0.2525	-8w650 to 9w155	No	ns	
	12h vs. 24h	-1.299	-10w20 to 7w603	No	ns	
**1.1.7.**	**300 µg/ml**					
	1h vs. 12h	4.45	-4w452 to 13w35	No	ns	
	1h vs. 24h	-0.0972	-8w999 to 8w805	No	ns	
	12h vs. 24h	-4.547	-13w45 to 4w355	No	ns	
**1.1.8.**	**400 µg/ml**					
	1h vs. 12h	0.9007	-8w001 to 9w803	No	ns	
	1h vs. 24h	-1.847	-10w75 to 7w055	No	ns	
	12h vs. 24h	-2.748	-11w65 to 6w154	No	ns	
**1.1.9.**	**500 µg/ml**					
	1h vs. 12h	2.698	-6w204 to 11w60	No	ns	
	1h vs. 24h	3.148	-5w754 to 12w05	No	ns	
	12h vs. 24h	0.4497	-8w452 to 9w352	No	ns	
**1.1.10.**	**600 µg/ml**					
	1h vs. 12h	0.4497	-8w452 to 9w352	No	ns	
	1h vs. 24h	-2.648	-11w55 to 6w254	No	ns	
	12h vs. 24h	-3.098	-12w00 to 5w804	No	ns	

2.	Immunomodulation in J774.A.1Cell line					
	**Two-way ANOVA**	Ordinary				
	Alpha	0.05				
	**Source of Variation**	**% of total variation**	**P value**	**P value summary**	**Significant?**	
	Interaction	38.70	< 0.0001	****	Yes	
	Row Factor	32.67	< 0.0001	****	Yes	
	Column Factor	27.41	< 0.0001	****	Yes	
	**ANOVA table**	**SS**	**DF**	**MS**	**F (DFn, DFd)**	**P value**
	Interaction	348.3	14	24.88	F (14, 48) = 108.2	P < 0.0001
	Row Factor	294.1	7	42.01	F (7, 48) = 182.7	P < 0.0001
	Column Factor	246.7	2	123.3	F (2, 48) = 536.3	P < 0.0001
	Residual	11.04	48	0.2300		
**2.1.**	**Tukey's multiple comparisons test**	**Mean Diffw**	**95% CI of diffw**	**Significant?**	**Summary**	
**2.1.1.**	**TLR2**					
	Mϕ vs. Mϕ + LPS	-0.4	-1.347 to 0.5470	No	ns	
	Mϕ vs. Mϕ + Furanone	-1.6	-2.547 to -0.6530	Yes	***	
	Mϕ + LPS vs. Mϕ + Furanone	-1.2	-2.147 to -0.2530	Yes	*	
**2.1.2.**	**IL-ß1**					
	Mϕ vs. Mϕ + LPS	-4.9	-5.847 to -3.953	Yes	****	
	Mϕ vs. Mϕ + Furanone	-5.2	-6.147 to -4.253	Yes	****	
	Mϕ + LPS vs. Mϕ + Furanone	-0.3	-1.247 to 0.6470	No	ns	
**2.1.3.**	**TNF-α**					
	Mϕ vs. Mϕ + LPS	-14	-14.95 to -13.05	Yes	****	
	Mϕ vs. Mϕ + Furanone	-4	-4.947 to -3.053	Yes	****	
	Mϕ + LPS vs. Mϕ + Furanone	10	9.053 to 10.95	Yes	****	
**2.1.4.**	**IL-6**					
	Mϕ vs. Mϕ + LPS	-6	-6.947 to -5.053	Yes	****	
	Mϕ vs. Mϕ + Furanone	0	-0.9470 to 0.9470	No	ns	
	Mϕ + LPS vs. Mϕ + Furanone	6	5.053 to 6.947	Yes	****	
**2.1.5.**	**IL-10**					
	Mϕ vs. Mϕ LPS	0.4000	-0.5470 to 1.347	No	ns	
	Mϕ vs. Mϕ Furanone	0.5000	-0.4470 to 1.447	No	ns	

	Mϕ LPS vs. Mϕ Furanone	0.1000	-0.8470 to 1.047	No	ns	
**2.1.6.**	**IL-12**					
	Mϕ vs. Mϕ + LPS	-1.9	-2.847 to -0.9530	Yes	****	
	Mϕ vs. Mϕ + Furanone	-0.3	-1.247 to 0.6470	No	ns	
	Mϕ + LPS vs. Mϕ + Furanone	1.6	0.6530 to 2.547	Yes	***	
**2.1.7.**	**IL-4**					
	Mϕ vs. Mϕ + LPS	0.3	-0.6470 to 1.247	No	ns	
	Mϕ vs. Mϕ + Furanone	0.1	-0.8470 to 1.047	No	ns	
	Mϕ + LPS vs. Mϕ + Furanone	-0.2	-1.147 to 0.7470	No	ns	
	**IFN-γ**					
**2.1.8.**	Mϕ vs. Mϕ + LPS	-9	-9.947 to -8.053	Yes	****	
	Mϕ vs. Mϕ + Furanone	-0.8	-1.747 to 0.1470	No	ns	
	Mϕ + LPS vs. Mϕ + Furanone	8.2	7.253 to 9.147	Yes	****	

Supplementary File S3: Two way ANOVA followed by Tukey's multiple comparisons test was performed for body weight and feed intake by BALB/c mice during treatment of cryptococcosis using compound-**6**.

**1.**	**Body Weight**					
	**Two-way ANOVA**	Ordinary				
	Alpha	0.05				
	**Source of Variation**	**% of total variation**	**P value**	**P value summary**	**Significant?**	
	Interaction	44.6	< 0.0001	****	Yes	
	Row Factor	31.26	< 0.0001	****	Yes	
	Column Factor	19.14	< 0.0001	****	Yes	
	**ANOVA table**	**SS**	**DF**	**MS**	**F (DFn, DFd)**	**P value**
	Interaction	1213	16	75.82	F (16, 50) = 27.86	P < 0.0001
	Row Factor	850.2	4	212.6	F (4, 50) = 78.09	P < 0.0001
	Column Factor	520.6	4	130.2	F (4, 50) = 47.82	P < 0.0001
	Residual	136.1	50	2.722		
**1.1.**	**Tukey's multiple comparisons test**	**Mean Diff.**	**95% CI of diff.**	**Significant?**	**Summary**	
**1.1.1.**	**Control**					
	Acclimatization Stage vs. Infection Day	5.95	2.138 to 9.762	Yes	***	
	Acclimatization Stage vs. Post infection Week I	5.89	2.078 to 9.702	Yes	***	
	Acclimatization Stage vs. Post infection Week II	5.89	2.078 to 9.702	Yes	***	
	Acclimatization Stage vs. Post infection Week III	7.69	3.878 to 11.50	Yes	****	
	Infection Day vs. Post infection Week I	-0.06	-3.872 to 3.752	No	ns	
	Infection Day vs. Post infection Week II	-0.06	-3.872 to 3.752	No	ns	
	Infection Day vs. Post infection Week III	1.74	-2.072 to 5.552	No	ns	
	Post infection Week I vs. Post infection Week II	0	-3.812 to 3.812	No	ns	
	Post infection Week I vs. Post infection Week III	1.8	-2.012 to 5.612	No	ns	
	Post infection Week II vs. Post infection Week III	1.8	-2.012 to 5.612	No	ns	
**1.1.2.**	**Disease Control**					
	Acclimatization Stage vs. Infection Day	2.05	-1.762 to 5.862	No	ns	
	Acclimatization Stage vs. Post infection Week I	2.67	-1.142 to 6.482	No	ns	
	Acclimatization Stage vs. Post infection Week II	14.33	10.52 to 18.14	Yes	****	
	Acclimatization Stage vs. Post infection Week III	29.41	25.60 to 33.22	Yes	****	
	Infection Day vs. Post infection Week I	0.62	-3.192 to 4.432	No	ns	
	Infection Day vs. Post infection Week II	12.28	8.468 to 16.09	Yes	****	
	Infection Day vs. Post infection Week III	27.36	23.55 to 31.17	Yes	****	
	Post infection Week I vs. Post infection Week II	11.66	7.848 to 15.47	Yes	****	
	Post infection Week I vs. Post infection Week III	26.74	22.93 to 30.55	Yes	****	

	Post infection Week II vs. Post infection Week III	15.08	11.27 to 18.89	Yes	****	
**1.1.3.**	**Drug Control**					
	Acclimatization Stage vs. Infection Day	2.41	-1.402 to 6.222	No	ns	
	Acclimatization Stage vs. Post infection Week I	2.84	-0.9720 to 6.652	No	ns	
	Acclimatization Stage vs. Post infection Week II	5.31	1.498 to 9.122	Yes	**	
	Acclimatization Stage vs. Post infection Week III	6.48	2.668 to 10.29	Yes	***	
	Infection Day vs. Post infection Week I	0.43	-3.382 to 4.242	No	ns	
	Infection Day vs. Post infection Week II	2.9	-0.9120 to 6.712	No	ns	
	Infection Day vs. Post infection Week III	4.07	0.2580 to 7.882	Yes	*	
	Post infection Week I vs. Post infection Week II	2.47	-1.342 to 6.282	No	ns	
	Post infection Week I vs. Post infection Week III	3.64	-0.1720 to 7.452	No	ns	
	Post infection Week II vs. Post infection Week III	1.17	-2.642 to 4.982	No	ns	
**1.1.4.**	**Low Dose**					
	Acclimatization Stage vs. Infection Day	2.04	-1.772 to 5.852	No	ns	
	Acclimatization Stage vs. Post infection Week I	2.43	-1.382 to 6.242	No	ns	
	Acclimatization Stage vs. Post infection Week II	2.62	-1.192 to 6.432	No	ns	
	Acclimatization Stage vs. Post infection Week III	2.22	-1.592 to 6.032	No	ns	
	Infection Day vs. Post infection Week I	0.39	-3.422 to 4.202	No	ns	
	Infection Day vs. Post infection Week II	0.58	-3.232 to 4.392	No	ns	
	Infection Day vs. Post infection Week III	0.18	-3.632 to 3.992	No	ns	
	Post infection Week I vs. Post infection Week II	0.19	-3.622 to 4.002	No	ns	
	Post infection Week I vs. Post infection Week III	-0.21	-4.022 to 3.602	No	ns	
	Post infection Week II vs. Post infection Week III	-0.4	-4.212 to 3.412	No	ns	
**1.1.4.**	**Effective Dose**					
	Acclimatization Stage vs. Infection Day	3	-0.8120 to 6.812	No	ns	
	Acclimatization Stage vs. Post infection Week I	3.46	-0.3520 to 7.272	No	ns	
	Acclimatization Stage vs. Post infection Week II	3.69	-0.1220 to 7.502	No	ns	
	Acclimatization Stage vs. Post infection Week III	4.04	0.2280 to 7.852	Yes	*	
	Infection Day vs. Post infection Week I	0.46	-3.352 to 4.272	No	ns	
	Infection Day vs. Post infection Week II	0.69	-3.122 to 4.502	No	ns	
	Infection Day vs. Post infection Week III	1.04	-2.772 to 4.852	No	ns	
	Post infection Week I vs. Post infection Week II	0.23	-3.582 to 4.042	No	ns	
	Post infection Week I vs. Post infection Week III	0.58	-3.232 to 4.392	No	ns	
	Post infection Week II vs. Post infection Week III	0.35	-3.462 to 4.162	No	ns	
**1.2.**	**Tukey's multiple comparisons test**	**Mean Diff.**	**95% CI of diff.**	**Significant?**	**Summary**	
	Control vs. Disease Control	1.758	0.05324 to 3.463	Yes	*	
	Control vs. Drug Control	-3.316	-5.021 to -1.611	Yes	****	

	Control vs. Low Dose	-4.972	-6.677 to -3.267	Yes	****	
	Control vs. Effective Dose	-4.496	-6.201 to -2.791	Yes	****	
	Disease Control vs. Drug Control	-5.074	-6.779 to -3.369	Yes	****	
	Disease Control vs. Low Dose	-6.73	-8.435 to -5.025	Yes	****	
	Disease Control vs. Effective Dose	-6.254	-7.959 to -4.549	Yes	****	
	Drug Control vs. Low Dose	-1.656	-3.361 to 0.04876	No	ns	
	Drug Control vs. Effective Dose	-1.18	-2.885 to 0.5248	No	ns	
	Low Dose vs. Effective Dose	0.476	-1.229 to 2.181	No	ns	

**2.**	**Feed Intake**					
	Two-way ANOVA	Ordinary				
	Alpha	0.05				
	**Source of Variation**	**% of total variation**	**P value**	**P value summary**	**Significant?**	
	Interaction	30.29	0.0001	***	Yes	
	Row Factor	32.67	< 0.0001	****	Yes	
	Column Factor	12.59	0.0003	***	Yes	
	**ANOVA table**	**SS**	**DF**	**MS**	**F (DFn, DFd)**	**P value**
	Interaction	338.2	16	21.14	F (16, 50) = 3.873	P = 0.0001
	Row Factor	364.7	4	91.18	F (4, 50) = 16.71	P < 0.0001
	Column Factor	140.6	4	35.15	F (4, 50) = 6.441	P = 0.0003
	Residual	272.9	50	5.458		
**2.1.**	**Tukey's multiple comparisons test**	**Mean Diff.**	**95% CI of diff.**	**Significant?**	**Summary**	
**2.1.1.**	**Control**					
	Acclimatization Stage vs. Infection Day	-1.69	-7.088 to 3.708	No	ns	
	Acclimatization Stage vs. Post infection Week I	-0.7	-6.098 to 4.698	No	ns	
	Acclimatization Stage vs. Post infection Week II	-1.27	-6.668 to 4.128	No	ns	
	Acclimatization Stage vs. Post infection Week III	3.08	-2.318 to 8.478	No	ns	
	Infection Day vs. Post infection Week I	0.99	-4.408 to 6.388	No	ns	
	Infection Day vs. Post infection Week II	0.42	-4.978 to 5.818	No	ns	
	Infection Day vs. Post infection Week III	4.77	-0.6279 to 10.17	No	ns	
	Post infection Week I vs. Post infection Week II	-0.57	-5.968 to 4.828	No	ns	
	Post infection Week I vs. Post infection Week III	3.78	-1.618 to 9.178	No	ns	
	Post infection Week II vs. Post infection Week III	4.35	-1.048 to 9.748	No	ns	
**2.1.2.**	**Disease Control**					
	Acclimatization Stage vs. Infection Day	0.25	-5.148 to 5.648	No	ns	
	Acclimatization Stage vs. Post infection Week I	4.26	-1.138 to 9.658	No	ns	
	Acclimatization Stage vs. Post infection Week II	-0.66	-6.058 to 4.738	No	ns	

	Acclimatization Stage vs. Post infection Week III	14.86	9.462 to 20.26	Yes	****	
	Infection Day vs. Post infection Week I	4.01	-1.388 to 9.408	No	ns	
	Infection Day vs. Post infection Week II	-0.91	-6.308 to 4.488	No	ns	
	Infection Day vs. Post infection Week III	14.61	9.212 to 20.01	Yes	****	
	Post infection Week I vs. Post infection Week II	-4.92	-10.32 to 0.4779	No	ns	
	Post infection Week I vs. Post infection Week III	10.6	5.202 to 16.00	Yes	****	
	Post infection Week II vs. Post infection Week III	15.52	10.12 to 20.92	Yes	****	
**2.1.3.**	**Drug Control**					
	Acclimatization Stage vs. Infection Day	-0.82	-6.218 to 4.578	No	ns	
	Acclimatization Stage vs. Post infection Week I	-0.76	-6.158 to 4.638	No	ns	
	Acclimatization Stage vs. Post infection Week II	-2.22	-7.618 to 3.178	No	ns	
	Acclimatization Stage vs. Post infection Week III	-0.05	-5.448 to 5.348	No	ns	
	Infection Day vs. Post infection Week I	0.06	-5.338 to 5.458	No	ns	
	Infection Day vs. Post infection Week II	-1.4	-6.798 to 3.998	No	ns	
	Infection Day vs. Post infection Week III	0.77	-4.628 to 6.168	No	ns	
	Post infection Week I vs. Post infection Week II	-1.46	-6.858 to 3.938	No	ns	
	Post infection Week I vs. Post infection Week III	0.71	-4.688 to 6.108	No	ns	
	Post infection Week II vs. Post infection Week III	2.17	-3.228 to 7.568	No	ns	
**2.1.4.**	**Low Dose**					
	Acclimatization Stage vs. Infection Day	-1.22	-6.618 to 4.178	No	ns	
	Acclimatization Stage vs. Post infection Week I	-0.21	-5.608 to 5.188	No	ns	
	Acclimatization Stage vs. Post infection Week II	-1.91	-7.308 to 3.488	No	ns	
	Acclimatization Stage vs. Post infection Week III	6.48	1.082 to 11.88	Yes	*	
	Infection Day vs. Post infection Week I	1.01	-4.388 to 6.408	No	ns	
	Infection Day vs. Post infection Week II	-0.69	-6.088 to 4.708	No	ns	
	Infection Day vs. Post infection Week III	7.7	2.302 to 13.10	Yes	**	
	Post infection Week I vs. Post infection Week II	-1.7	-7.098 to 3.698	No	ns	
	Post infection Week I vs. Post infection Week III	6.69	1.292 to 12.09	Yes	**	
	Post infection Week II vs. Post infection Week III	8.39	2.992 to 13.79	Yes	***	
**2.1.5.**	**Effective Dose**					
	Acclimatization Stage vs. Infection Day	1.47	-3.928 to 6.868	No	ns	
	Acclimatization Stage vs. Post infection Week I	0.9	-4.498 to 6.298	No	ns	
	Acclimatization Stage vs. Post infection Week II	-0.3	-5.698 to 5.098	No	ns	
	Acclimatization Stage vs. Post infection Week III	0.8	-4.598 to 6.198	No	ns	
	Infection Day vs. Post infection Week I	-0.57	-5.968 to 4.828	No	ns	
	Infection Day vs. Post infection Week II	-1.77	-7.168 to 3.628	No	ns	
	Infection Day vs. Post infection Week III	-0.67	-6.068 to 4.728	No	ns	
	Post infection Week I vs. Post infection Week II	-1.2	-6.598 to 4.198	No	ns	

	Post infection Week I vs. Post infection Week III	-0.1	-5.498 to 5.298	No	ns	
	Post infection Week II vs. Post infection Week III	1.1	-4.298 to 6.498	No	ns	
**2.2.**	**Tukey's multiple comparisons test**	**Mean Diff.**	**95% CI of diff.**	**Significant?**	**Summary**	
	Control vs. Disease Control	2.268	-0.1460 to 4.682	No	ns	
	Control vs. Drug Control	-0.664	-3.078 to 1.750	No	ns	
	Control vs. Low Dose	-1.746	-4.160 to 0.6680	No	ns	
	Control vs. Effective Dose	0.93	-1.484 to 3.344	No	ns	
	Disease Control vs. Drug Control	-2.932	-5.346 to -0.5180	Yes	*	
	Disease Control vs. Low Dose	-4.014	-6.428 to -1.600	Yes	***	
	Disease Control vs. Effective Dose	-1.338	-3.752 to 1.076	No	ns	
	Drug Control vs. Low Dose	-1.082	-3.496 to 1.332	No	ns	
	Drug Control vs. Effective Dose	1.594	-0.8200 to 4.008	No	ns	
	Low Dose vs. Effective Dose	2.676	0.2620 to 5.090	Yes	*	

Supplementary File S4: One way and two way ANOVA followed by Tukey's multiple comparisons test was performed for biodistribution of drug and fungal burden in different organs of BALB/c mice during treatment of cryptococcosis using compound-**6**.

**1.**	**Biodistribution of Drug**					
**1.1.**	**One Way ANOVA summary**					
	F	20.21				
	P value	0.0005				
	P value summary	***				
	Are differences among means statistically significant? (P < 0.05)	Yes				
	R square	0.8179				
**1.2.**	**ANOVA table**	**SS**	**DF**	**MS**	**F (DFn, DFd)**	**P value**
	Treatment (between columns)	36998	2	18499	F (2, 9) = 20.21	P = 0.0005
	Residual (within columns)	8237	9	915.2		
	Total	45235	11			
**1.3.**	**Tukey's multiple comparisons test**	**Mean Diff.**	**95% CI of diff.**	**Significant?**	**Summary**	
**1.3.1.**	**Brain**					
	Drug Control vs. Low Dose	3.1	-84.50 to 90.70	No	ns	
	Drug Control vs. Effective Dose	-94.7	-182.3 to -7.094	Yes	*	
	Low Dose vs. Effective Dose	-97.8	-185.4 to -10.19	Yes	*	
**1.3.2.**	**Lungs**					
	Drug Control vs. Low Dose	1.999	-85.60 to 89.60	No	ns	
	Drug Control vs. Effective Dose	-191.7	-279.3 to -104.1	Yes	****	
	Low Dose vs. Effective Dose	-193.7	-281.3 to -106.1	Yes	****	
**1.3.3.**	**Liver**					
	Drug Control vs. Low Dose	15.27	-72.33 to 102.9	No	ns	
	Drug Control vs. Effective Dose	-59	-146.6 to 28.60	No	ns	
	Low Dose vs. Effective Dose	-74.27	-161.9 to 13.33	No	ns	
**1.3.4.**	**Kidney**					
	Drug Control vs. Low Dose	14.36	-73.24 to 102.0	No	ns	
	Drug Control vs. Effective Dose	-107.4	-195.0 to -19.82	Yes	*	
	Low Dose vs. Effective Dose	-121.8	-209.4 to -34.19	Yes	**	

**2.**	**Fungal Burden**					
**2.1.**	**Two-way ANOVA**	**Ordinary**				
	Alpha	0.05				

	Source of Variation	% of total variation	P value	P value summary	Significant?	
	Interaction	16.99	< 0.0001	****	Yes	
	Row Factor	6.813	< 0.0001	****	Yes	
	Column Factor	71.46	< 0.0001	****	Yes	
**2.2.**	**ANOVA table**	**SS**	**DF**	**MS**	**F (DFn, DFd)**	**P value**
	Interaction	2.7E+09	12	2E+08	F (12, 40) = 11.96	P < 0.0001
	Row Factor	1.1E+09	3	4E+08	F (3, 40) = 19.19	P < 0.0001
	Column Factor	1.1E+10	4	3E+09	F (4, 40) = 151.0	P < 0.0001
	Residual	7.6E+08	40	2E+07		
**2.3.**	**Tukey's multiple comparisons test**	**Mean Diff.**	**95% CI of diff.**	**Significant?**	**Summary**	
**2.3.1.**	**Brain**					
	Control vs. Disease Control	-32800	-42933 to -22667	Yes	****	
	Control vs. Drug Control	-30850	-40983 to -20717	Yes	****	
	Control vs. Low Dose	-34600	-44733 to -24467	Yes	****	
	Control vs. Effective Dose	0	-10133 to 10133	No	ns	
	Disease Control vs. Drug Control	1950	-8183 to 12083	No	ns	
	Disease Control vs. Low Dose	-1800	-11933 to 8333	No	ns	
	Disease Control vs. Effective Dose	32800	22667 to 42933	Yes	****	
	Drug Control vs. Low Dose	-3750	-13883 to 6383	No	ns	
	Drug Control vs. Effective Dose	30850	20717 to 40983	Yes	****	
	Low Dose vs. Effective Dose	34600	24467 to 44733	Yes	****	
**2.3.2.**	**Lungs**					
	Control vs. Disease Control	-51500	-61633 to -41367	Yes	****	
	Control vs. Drug Control	-300	-10433 to 9833	No	ns	
	Control vs. Low Dose	-27100	-37233 to -16967	Yes	****	
	Control vs. Effective Dose	-500	-10633 to 9633	No	ns	
	Disease Control vs. Drug Control	51200	41067 to 61333	Yes	****	
	Disease Control vs. Low Dose	24400	14267 to 34533	Yes	****	
	Disease Control vs. Effective Dose	51000	40867 to 61133	Yes	****	
	Drug Control vs. Low Dose	-26800	-36933 to -16667	Yes	****	
	Drug Control vs. Effective Dose	-200	-10333 to 9933	No	ns	
	Low Dose vs. Effective Dose	26600	16467 to 36733	Yes	****	
**2.3.3.**	**Liver**					
	Control vs. Disease Control	-24900	-35033 to -14767	Yes	****	
	Control vs. Drug Control	-3200	-13333 to 6933	No	ns	
	Control vs. Low Dose	-12750	-22883 to -2617	Yes	**	

	Control vs. Effective Dose	0	-10133 to 10133	No	ns	
	Disease Control vs. Drug Control	21700	11567 to 31833	Yes	****	
	Disease Control vs. Low Dose	12150	2017 to 22283	Yes	*	
	Disease Control vs. Effective Dose	24900	14767 to 35033	Yes	****	
	Drug Control vs. Low Dose	-9550	-19683 to 583.4	No	ns	
	Drug Control vs. Effective Dose	3200	-6933 to 13333	No	ns	
	Low Dose vs. Effective Dose	12750	2617 to 22883	Yes	**	
**2.3.4.**	**Kidney**					
	Control vs. Disease Control	-28350	-38483 to -18217	Yes	****	
	Control vs. Drug Control	-5750	-15883 to 4383	No	ns	
	Control vs. Low Dose	-27200	-37333 to -17067	Yes	****	
	Control vs. Effective Dose	0	-10133 to 10133	No	ns	
	Disease Control vs. Drug Control	22600	12467 to 32733	Yes	****	
	Disease Control vs. Low Dose	1150	-8983 to 11283	No	ns	
	Disease Control vs. Effective Dose	28350	18217 to 38483	Yes	****	
	Drug Control vs. Low Dose	-21450	-31583 to -11317	Yes	****	
	Drug Control vs. Effective Dose	5750	-4383 to 15883	No	ns	
	Low Dose vs. Effective Dose	27200	17067 to 37333	Yes	****	

Supplementary File S5: Histopathology score

PARAMETER		Groups	Control	Control	Disease control	Disease control	Drug Control	Drug Control	Low dose	Low dose	Effective Dose	Effective Dose
		**Animal Number**	**16-7863**	**16-7864**	**16-7857**	**16-7858**	**16-7852**	**16-7853**	**16-7872**	**16-7874**	**16-7867**	**16-7868**
1	Histiocytic Reaction		1	2	5	5	1	1	2	4	1	1
2	Lympho-plasmacytic infiltration		2	2	3	3	1	1	2	3	0	1
3	Neutrophilic infiltration		0	3	5	5	1	0	2	4	0	1
4	Expansion of the alveolar space		0	0	0	2	0	0	2	1	0	1
5	Collapse of the alveolar lumen		1	1	3	2	0	0	2	3	0	1
6	Proliferation of yeast		0	0	3	4	0	0	1	4	0	0
7	Congestion / haemorrhage		1	2	2	2	1	1	1	4	1	1
	**Cumulative Total**		**5**	**10**	**21**	**23**	**4**	**3**	**12**	**23**	**2**	**6**

Overall histopathology scoring

	Control	Disease control	Standard	Low dose	High Dose
**Animal 1**	5	21	4	12	2
**Animal 2**	10	23	3	23	6
**Average ± SEM**	**7.5 ± 2.50**	**22 ± 0.81**	**3.5 ± 0.40**	**17.5 ± 5.50**	**4.0 ± 1.63**

Supplementary Table S6: Two way ANOVA followed by Tukey's multiple comparisons test was performed for histopathology score in different organs of BALB/c mice during treatment of cryptococcosis using compound-**6**.

**Histopathological Score**					
**Two-way ANOVA**	Ordinary				
Alpha	0.05				
**Source of Variation**	**% of total variation**	**P value**	**P value summary**	**Significant?**	
Interaction	26.34	< 0.0001	****	Yes	
Row Factor	52.34	< 0.0001	****	Yes	
Column Factor	14.79	< 0.0001	****	Yes	
**ANOVA table**	**SS**	**DF**	**MS**	**F (DFn, DFd)**	**P value**
Interaction	601.5	28	21.48	F (28, 80) = 11.51	P < 0.0001
Row Factor	1195	7	170.7	F (7, 80) = 91.51	P < 0.0001
Column Factor	337.7	4	84.42	F (4, 80) = 45.25	P < 0.0001
Residual	149.3	80	1.866		
**Tukey's multiple comparisons test**	**Mean Diff.**	**95% CI of diff.**	**Significant?**	**Summary**	
**Histiocytic Reaction**					
Control vs. Disease control	-2.5	-5.613 to 0.6128	No	ns	
Control vs. Drug Control	0.5	-2.613 to 3.613	No	ns	
Control vs. Low Dose	-1.5	-4.613 to 1.613	No	ns	
Control vs. Effective Dose	0.5	-2.613 to 3.613	No	ns	
Disease control vs. Drug Control	3	-0.1128 to 6.113	No	ns	
Disease control vs. Low Dose	1	-2.113 to 4.113	No	ns	
Disease control vs. Effective Dose	3	-0.1128 to 6.113	No	ns	
Drug Control vs. Low Dose	-2	-5.113 to 1.113	No	ns	
Drug Control vs. Effective Dose	0	-3.113 to 3.113	No	ns	
Low Dose vs. Effective Dose	2	-1.113 to 5.113	No	ns	
**Lympho-plasmacytic infiltration**					
Control vs. Disease control	0.33	-2.783 to 3.443	No	ns	
Control vs. Drug Control	1	-2.113 to 4.113	No	ns	
Control vs. Low Dose	-0.5	-3.613 to 2.613	No	ns	
Control vs. Effective Dose	1.33	-1.783 to 4.443	No	ns	
Disease control vs. Drug Control	0.67	-2.443 to 3.783	No	ns	
Disease control vs. Low Dose	-0.83	-3.943 to 2.283	No	ns	
Disease control vs. Effective Dose	1	-2.113 to 4.113	No	ns	
Drug Control vs. Low Dose	-1.5	-4.613 to 1.613	No	ns	
Drug Control vs. Effective Dose	0.33	-2.783 to 3.443	No	ns	

Low Dose vs. Effective Dose	1.83	-1.283 to 4.943	No	ns	
**Neutrophilic infiltration**					
Control vs. Disease control	-2.17	-5.283 to 0.9428	No	ns	
Control vs. Drug Control	0.83	-2.283 to 3.943	No	ns	
Control vs. Low Dose	-1.5	-4.613 to 1.613	No	ns	
Control vs. Effective Dose	0.83	-2.283 to 3.943	No	ns	
Disease control vs. Drug Control	3	-0.1128 to 6.113	No	ns	
Disease control vs. Low Dose	0.67	-2.443 to 3.783	No	ns	
Disease control vs. Effective Dose	3	-0.1128 to 6.113	No	ns	
Drug Control vs. Low Dose	-2.33	-5.443 to 0.7828	No	ns	
Drug Control vs. Effective Dose	0	-3.113 to 3.113	No	ns	
Low Dose vs. Effective Dose	2.33	-0.7828 to 5.443	No	ns	
**Expansion of the alveolar space**					
Control vs. Disease control	-1.33	-4.443 to 1.783	No	ns	
Control vs. Drug Control	-0.33	-3.443 to 2.783	No	ns	
Control vs. Low Dose	-1	-4.113 to 2.113	No	ns	
Control vs. Effective Dose	-0.33	-3.443 to 2.783	No	ns	
Disease control vs. Drug Control	1	-2.113 to 4.113	No	ns	
Disease control vs. Low Dose	0.33	-2.783 to 3.443	No	ns	
Disease control vs. Effective Dose	1	-2.113 to 4.113	No	ns	
Drug Control vs. Low Dose	-0.67	-3.783 to 2.443	No	ns	
Drug Control vs. Effective Dose	0	-3.113 to 3.113	No	ns	
Low Dose vs. Effective Dose	0.67	-2.443 to 3.783	No	ns	
**Collapse of the alveolar lumen**					
Control vs. Disease control	-0.67	-3.783 to 2.443	No	ns	
Control vs. Drug Control	0.67	-2.443 to 3.783	No	ns	
Control vs. Low Dose	-1.5	-4.613 to 1.613	No	ns	
Control vs. Effective Dose	0.33	-2.783 to 3.443	No	ns	
Disease control vs. Drug Control	1.34	-1.773 to 4.453	No	ns	
Disease control vs. Low Dose	-0.83	-3.943 to 2.283	No	ns	
Disease control vs. Effective Dose	1	-2.113 to 4.113	No	ns	
Drug Control vs. Low Dose	-2.17	-5.283 to 0.9428	No	ns	
Drug Control vs. Effective Dose	-0.34	-3.453 to 2.773	No	ns	
Low Dose vs. Effective Dose	1.83	-1.283 to 4.943	No	ns	
**Proliferation of yeast**					
Control vs. Disease control	-3	-6.113 to 0.1128	No	ns	
Control vs. Drug Control	0	-3.113 to 3.113	No	ns	

Control vs. Low Dose	-2.5	-5.613 to 0.6128	No	ns	
Control vs. Effective Dose	0	-3.113 to 3.113	No	ns	
Disease control vs. Drug Control	3	-0.1128 to 6.113	No	ns	
Disease control vs. Low Dose	0.5	-2.613 to 3.613	No	ns	
Disease control vs. Effective Dose	3	-0.1128 to 6.113	No	ns	
Drug Control vs. Low Dose	-2.5	-5.613 to 0.6128	No	ns	
Drug Control vs. Effective Dose	0	-3.113 to 3.113	No	ns	
Low Dose vs. Effective Dose	2.5	-0.6128 to 5.613	No	ns	
**Congestion / haemorrhage**					
Control vs. Disease control	-0.83	-3.943 to 2.283	No	ns	
Control vs. Drug Control	0.5	-2.613 to 3.613	No	ns	
Control vs. Low Dose	-1	-4.113 to 2.113	No	ns	
Control vs. Effective Dose	0.5	-2.613 to 3.613	No	ns	
Disease control vs. Drug Control	1.33	-1.783 to 4.443	No	ns	
Disease control vs. Low Dose	-0.17	-3.283 to 2.943	No	ns	
Disease control vs. Effective Dose	1.33	-1.783 to 4.443	No	ns	
Drug Control vs. Low Dose	-1.5	-4.613 to 1.613	No	ns	
Drug Control vs. Effective Dose	0	-3.113 to 3.113	No	ns	
Low Dose vs. Effective Dose	1.5	-1.613 to 4.613	No	ns	
**Overall histopathology scoring**					
Control vs. Disease control	-14.5	-17.61 to -11.39	Yes	****	
Control vs. Drug Control	4	0.8872 to 7.113	Yes	**	
Control vs. Low Dose	-10	-13.11 to -6.887	Yes	****	
Control vs. Effective Dose	3.5	0.3872 to 6.613	Yes	*	
Disease control vs. Drug Control	18.5	15.39 to 21.61	Yes	****	
Disease control vs. Low Dose	4.5	1.387 to 7.613	Yes	**	
Disease control vs. Effective Dose	18	14.89 to 21.11	Yes	****	
Drug Control vs. Low Dose	-14	-17.11 to -10.89	Yes	****	
Drug Control vs. Effective Dose	-0.5	-3.613 to 2.613	No	ns	
Low Dose vs. Effective Dose	13.5	10.39 to 16.61	Yes	****	

Supplementary Table S7: Two way ANOVA followed by Tukey's multiple comparisons test was performed for gene expression in Lungs and Brain of BALB/c mice during treatment of cryptococcosis using compound-**6**.

**1.**	**Lungs**					
1.1.	**Two-way ANOVA**	Ordinary				
	Alpha	0.05				
	**Source of Variation**	**% of total variation**	**P value**	**P value summary**	**Significant?**	
	Interaction	56.37	< 0.0001	****	Yes	
	Row Factor	24.41	< 0.0001	****	Yes	
	Column Factor	18.74	< 0.0001	****	Yes	
	**ANOVA table**	**SS**	**DF**	**MS**	**F (DFn, DFd)**	**P value**
	Interaction	1877	28	67.04	F (28, 80) = 339.8	P < 0.0001
	Row Factor	812.8	7	116.1	F (7, 80) = 588.5	P < 0.0001
	Column Factor	624	4	156	F (4, 80) = 790.6	P < 0.0001
	Residual	15.79	80	0.197		
**1.2.**	**Tukey's multiple comparisons test**	**Mean Diff.**	**95% CI of diff.**	**Significant?**	**Summary**	
**1.2.1.**	**TLR-2**					
	Control vs. Disease control	-8.83	-10.30 to -7.364	Yes	****	
	Control vs. Drug Control	0.23	-1.236 to 1.696	No	ns	
	Control vs. Low Dose	0.19	-1.276 to 1.656	No	ns	
	Control vs. Effective Dose	0.75	-0.7160 to 2.216	No	ns	
	Disease control vs. Drug Control	9.06	7.594 to 10.53	Yes	****	
	Disease control vs. Low Dose	9.02	7.554 to 10.49	Yes	****	
	Disease control vs. Effective Dose	9.58	8.114 to 11.05	Yes	****	
	Drug Control vs. Low Dose	-0.04	-1.506 to 1.426	No	ns	
	Drug Control vs. Effective Dose	0.52	-0.9460 to 1.986	No	ns	
	Low Dose vs. Effective Dose	0.56	-0.9060 to 2.026	No	ns	
**1.2.2.**	**IL-ß1**					
	Control vs. Disease control	-26.74	-28.21 to -25.27	Yes	****	
	Control vs. Drug Control	0.67	-0.7960 to 2.136	No	ns	
	Control vs. Low Dose	-11.29	-12.76 to -9.824	Yes	****	
	Control vs. Effective Dose	0.28	-1.186 to 1.746	No	ns	
	Disease control vs. Drug Control	27.41	25.94 to 28.88	Yes	****	
	Disease control vs. Low Dose	15.45	13.98 to 16.92	Yes	****	
	Disease control vs. Effective Dose	27.02	25.55 to 28.49	Yes	****	
	Drug Control vs. Low Dose	-11.96	-13.43 to -10.49	Yes	****	

	Drug Control vs. Effective Dose	-0.39	-1.856 to 1.076	No	ns	
	Low Dose vs. Effective Dose	11.57	10.10 to 13.04	Yes	****	
**1.2.3.**	**TNF-a**					
	Control vs. Disease control	-3.98	-5.446 to -2.514	Yes	****	
	Control vs. Drug Control	-0.64	-2.106 to 0.8260	No	ns	
	Control vs. Low Dose	-15.32	-16.79 to -13.85	Yes	****	
	Control vs. Effective Dose	-0.01	-1.476 to 1.456	No	ns	
	Disease control vs. Drug Control	3.34	1.874 to 4.806	Yes	****	
	Disease control vs. Low Dose	-11.34	-12.81 to -9.874	Yes	****	
	Disease control vs. Effective Dose	3.97	2.504 to 5.436	Yes	****	
	Drug Control vs. Low Dose	-14.68	-16.15 to -13.21	Yes	****	
	Drug Control vs. Effective Dose	0.63	-0.8360 to 2.096	No	ns	
	Low Dose vs. Effective Dose	15.31	13.84 to 16.78	Yes	****	
**1.2.4.**	**IL-6**					
	Control vs. Disease control	-4.96	-6.426 to -3.494	Yes	****	
	Control vs. Drug Control	-0.17	-1.636 to 1.296	No	ns	
	Control vs. Low Dose	-0.08	-1.546 to 1.386	No	ns	
	Control vs. Effective Dose	-0.75	-2.216 to 0.7160	No	ns	
	Disease control vs. Drug Control	4.79	3.324 to 6.256	Yes	****	
	Disease control vs. Low Dose	4.88	3.414 to 6.346	Yes	****	
	Disease control vs. Effective Dose	4.21	2.744 to 5.676	Yes	****	
	Drug Control vs. Low Dose	0.09	-1.376 to 1.556	No	ns	
	Drug Control vs. Effective Dose	-0.58	-2.046 to 0.8860	No	ns	
	Low Dose vs. Effective Dose	-0.67	-2.136 to 0.7960	No	ns	
**1.2.5.**	**IL-10**					
	Control vs. Disease control	0.98	-0.4860 to 2.446	No	ns	
	Control vs. Drug Control	0.98	-0.4860 to 2.446	No	ns	
	Control vs. Low Dose	0.99	-0.4760 to 2.456	No	ns	
	Control vs. Effective Dose	0.96	-0.5060 to 2.426	No	ns	
	Disease control vs. Drug Control	-1E-08	-1.466 to 1.466	No	ns	
	Disease control vs. Low Dose	0.01	-1.456 to 1.476	No	ns	
	Disease control vs. Effective Dose	-0.02	-1.486 to 1.446	No	ns	
	Drug Control vs. Low Dose	0.01	-1.456 to 1.476	No	ns	
	Drug Control vs. Effective Dose	-0.02	-1.486 to 1.446	No	ns	
	Low Dose vs. Effective Dose	-0.03	-1.496 to 1.436	No	ns	
**1.2.6.**	**IL-12**					
	Control vs. Disease control	-1.66	-3.126 to -0.1940	Yes	**	

	Control vs. Drug Control	0.01	-1.456 to 1.476	No	ns	
	Control vs. Low Dose	-0.12	-1.586 to 1.346	No	ns	
	Control vs. Effective Dose	-0.91	-2.376 to 0.5560	No	ns	
	Disease control vs. Drug Control	1.67	0.2040 to 3.136	Yes	**	
	Disease control vs. Low Dose	1.54	0.07398 to 3.006	Yes	*	
	Disease control vs. Effective Dose	0.75	-0.7160 to 2.216	No	ns	
	Drug Control vs. Low Dose	-0.13	-1.596 to 1.336	No	ns	
	Drug Control vs. Effective Dose	-0.92	-2.386 to 0.5460	No	ns	
	Low Dose vs. Effective Dose	-0.79	-2.256 to 0.6760	No	ns	
**1.2.7.**	**IL-4**					
	Control vs. Disease control	-0.82	-2.286 to 0.6460	No	ns	
	Control vs. Drug Control	-0.18	-1.646 to 1.286	No	ns	
	Control vs. Low Dose	0.56	-0.9060 to 2.026	No	ns	
	Control vs. Effective Dose	-0.42	-1.886 to 1.046	No	ns	
	Disease control vs. Drug Control	0.64	-0.8260 to 2.106	No	ns	
	Disease control vs. Low Dose	1.38	-0.08602 to 2.846	No	ns	
	Disease control vs. Effective Dose	0.4	-1.066 to 1.866	No	ns	
	Drug Control vs. Low Dose	0.74	-0.7260 to 2.206	No	ns	
	Drug Control vs. Effective Dose	-0.24	-1.706 to 1.226	No	ns	
	Low Dose vs. Effective Dose	-0.98	-2.446 to 0.4860	No	ns	
**1.2.8.**	**IFN-g**					
	Control vs. Disease control	0.93	-0.5360 to 2.396	No	ns	
	Control vs. Drug Control	0.97	-0.4960 to 2.436	No	ns	
	Control vs. Low Dose	0.99	-0.4760 to 2.456	No	ns	
	Control vs. Effective Dose	-1.7	-3.166 to -0.2340	Yes	**	
	Disease control vs. Drug Control	0.04	-1.426 to 1.506	No	ns	
	Disease control vs. Low Dose	0.06	-1.406 to 1.526	No	ns	
	Disease control vs. Effective Dose	-2.63	-4.096 to -1.164	Yes	****	
	Drug Control vs. Low Dose	0.02	-1.446 to 1.486	No	ns	
	Drug Control vs. Effective Dose	-2.67	-4.136 to -1.204	Yes	****	
	Low Dose vs. Effective Dose	-2.69	-4.156 to -1.224	Yes	****	
**2.**	**Brain**					
**2.1.**	**Two-way ANOVA**	Ordinary				
	Alpha	0.05				
	**Source of Variation**	**% of total variation**	**P value**	**P value summary**	**Significant?**	
	Interaction	65.35	< 0.0001	****	Yes	
	Row Factor	20.48	< 0.0001	****	Yes	

	Column Factor	10.59	< 0.0001	****	Yes	
	**ANOVA table**	**SS**	**DF**	**MS**	**F (DFn, DFd)**	**P value**
	Interaction	309.2	28	11.04	F (28, 80) = 52.14	P < 0.0001
	Row Factor	96.87	7	13.84	F (7, 80) = 65.35	P < 0.0001
	Column Factor	50.09	4	12.52	F (4, 80) = 59.13	P < 0.0001
	Residual	16.94	80	0.212		
**2.2.**	**Tukey's multiple comparisons test**	**Mean Diff.**	**95% CI of diff.**	**Significant?**	**Summary**	
**2.2.1.**	**TLR-2**					
	Control vs. Disease control	-0.16	-1.209 to 0.8887	No	ns	
	Control vs. Drug Control	0.43	-0.6187 to 1.479	No	ns	
	Control vs. Low Dose	-11.19	-12.24 to -10.14	Yes	****	
	Control vs. Effective Dose	-1.28	-2.329 to -0.2313	Yes	**	
	Disease control vs. Drug Control	0.59	-0.4587 to 1.639	No	ns	
	Disease control vs. Low Dose	-11.03	-12.08 to -9.981	Yes	****	
	Disease control vs. Effective Dose	-1.12	-2.169 to -0.07134	Yes	*	
	Drug Control vs. Low Dose	-11.62	-12.67 to -10.57	Yes	****	
	Drug Control vs. Effective Dose	-1.71	-2.759 to -0.6613	Yes	***	
	Low Dose vs. Effective Dose	9.91	8.861 to 10.96	Yes	****	
**2.2.2.**	**IL-ß1**					
	Control vs. Disease control	0.63	-0.4187 to 1.679	No	ns	
	Control vs. Drug Control	0.57	-0.4787 to 1.619	No	ns	
	Control vs. Low Dose	-2.2	-3.249 to -1.151	Yes	****	
	Control vs. Effective Dose	0.22	-0.8287 to 1.269	No	ns	
	Disease control vs. Drug Control	-0.06	-1.109 to 0.9887	No	ns	
	Disease control vs. Low Dose	-2.83	-3.879 to -1.781	Yes	****	
	Disease control vs. Effective Dose	-0.41	-1.459 to 0.6387	No	ns	
	Drug Control vs. Low Dose	-2.77	-3.819 to -1.721	Yes	****	
	Drug Control vs. Effective Dose	-0.35	-1.399 to 0.6987	No	ns	
	Low Dose vs. Effective Dose	2.42	1.371 to 3.469	Yes	****	
**2.2.3.**	**TNF-a**					
	Control vs. Disease control	-0.54	-1.589 to 0.5087	No	ns	
	Control vs. Drug Control	-0.36	-1.409 to 0.6887	No	ns	
	Control vs. Low Dose	0.31	-0.7387 to 1.359	No	ns	
	Control vs. Effective Dose	0.36	-0.6887 to 1.409	No	ns	
	Disease control vs. Drug Control	0.18	-0.8687 to 1.229	No	ns	
	Disease control vs. Low Dose	0.85	-0.1987 to 1.899	No	ns	
	Disease control vs. Effective Dose	0.9	-0.1487 to 1.949	No	ns	

	Drug Control vs. Low Dose	0.67	-0.3787 to 1.719	No	ns	
	Drug Control vs. Effective Dose	0.72	-0.3287 to 1.769	No	ns	
	Low Dose vs. Effective Dose	0.05	-0.9987 to 1.099	No	ns	
**2.2.4.**	**IL-6**					
	Control vs. Disease control	-0.74	-1.789 to 0.3087	No	ns	
	Control vs. Drug Control	-2.09	-3.139 to -1.041	Yes	****	
	Control vs. Low Dose	-2.93	-3.979 to -1.881	Yes	****	
	Control vs. Effective Dose	-0.77	-1.819 to 0.2787	No	ns	
	Disease control vs. Drug Control	-1.35	-2.399 to -0.3013	Yes	**	
	Disease control vs. Low Dose	-2.19	-3.239 to -1.141	Yes	****	
	Disease control vs. Effective Dose	-0.03	-1.079 to 1.019	No	ns	
	Drug Control vs. Low Dose	-0.84	-1.889 to 0.2087	No	ns	
	Drug Control vs. Effective Dose	1.32	0.2713 to 2.369	Yes	**	
	Low Dose vs. Effective Dose	2.16	1.111 to 3.209	Yes	****	
**2.2.5.**	**IL-10**					
	Control vs. Disease control	0.8	-0.2487 to 1.849	No	ns	
	Control vs. Drug Control	0.85	-0.1987 to 1.899	No	ns	
	Control vs. Low Dose	1.02	-0.02866 to 2.069	No	ns	
	Control vs. Effective Dose	0.68	-0.3687 to 1.729	No	ns	
	Disease control vs. Drug Control	0.05	-0.9987 to 1.099	No	ns	
	Disease control vs. Low Dose	0.22	-0.8287 to 1.269	No	ns	
	Disease control vs. Effective Dose	-0.12	-1.169 to 0.9287	No	ns	
	Drug Control vs. Low Dose	0.17	-0.8787 to 1.219	No	ns	
	Drug Control vs. Effective Dose	-0.17	-1.219 to 0.8787	No	ns	
	Low Dose vs. Effective Dose	-0.34	-1.389 to 0.7087	No	ns	
**2.2.6.**	**IL-12**					
	Control vs. Disease control	0.15	-0.8987 to 1.199	No	ns	
	Control vs. Drug Control	-0.53	-1.579 to 0.5187	No	ns	
	Control vs. Low Dose	0.49	-0.5587 to 1.539	No	ns	
	Control vs. Effective Dose	-0.04	-1.089 to 1.009	No	ns	
	Disease control vs. Drug Control	-0.68	-1.729 to 0.3687	No	ns	
	Disease control vs. Low Dose	0.34	-0.7087 to 1.389	No	ns	
	Disease control vs. Effective Dose	-0.19	-1.239 to 0.8587	No	ns	
	Drug Control vs. Low Dose	1.02	-0.02866 to 2.069	No	ns	
	Drug Control vs. Effective Dose	0.49	-0.5587 to 1.539	No	ns	
	Low Dose vs. Effective Dose	-0.53	-1.579 to 0.5187	No	ns	
**2.2.7.**	**IL-4**					

	Control vs. Disease control	-0.37	-1.419 to 0.6787	No	ns	
	Control vs. Drug Control	-1.39	-2.439 to -0.3413	Yes	**	
	Control vs. Low Dose	0.3	-0.7487 to 1.349	No	ns	
	Control vs. Effective Dose	-2.45	-3.499 to -1.401	Yes	****	
	Disease control vs. Drug Control	-1.02	-2.069 to 0.02866	No	ns	
	Disease control vs. Low Dose	0.67	-0.3787 to 1.719	No	ns	
	Disease control vs. Effective Dose	-2.08	-3.129 to -1.031	Yes	****	
	Drug Control vs. Low Dose	1.69	0.6413 to 2.739	Yes	***	
	Drug Control vs. Effective Dose	-1.06	-2.109 to -0.01134	Yes	*	
	Low Dose vs. Effective Dose	-2.75	-3.799 to -1.701	Yes	****	
**2.2.8.**	**IFN-g**					
	Control vs. Disease control	-0.2	-1.249 to 0.8487	No	ns	
	Control vs. Drug Control	0.15	-0.8987 to 1.199	No	ns	
	Control vs. Low Dose	0.16	-0.8887 to 1.209	No	ns	
	Control vs. Effective Dose	-2.32	-3.369 to -1.271	Yes	****	
	Disease control vs. Drug Control	0.35	-0.6987 to 1.399	No	ns	
	Disease control vs. Low Dose	0.36	-0.6887 to 1.409	No	ns	
	Disease control vs. Effective Dose	-2.12	-3.169 to -1.071	Yes	****	
	Drug Control vs. Low Dose	0.01	-1.039 to 1.059	No	ns	
	Drug Control vs. Effective Dose	-2.47	-3.519 to -1.421	Yes	****	
	Low Dose vs. Effective Dose	-2.48	-3.529 to -1.431	Yes	****	
